# New Monoterpenoid Indoles with Osteoclast Activities from *Gelsemium elegans*

**DOI:** 10.3390/molecules26247457

**Published:** 2021-12-09

**Authors:** Xin Wei, Rui Guo, Xiao Wang, Jia-Jun Liang, Hao-Fei Yu, Cai-Feng Ding, Ting-Ting Feng, Li-Yan Zhang, Xia Liu, Xin-Yue Hu, Ying Zhou

**Affiliations:** 1 School of Pharmacy, Guizhou University of Traditional Chinese Medicine, Guiyang 550025, China; sfweixin@163.com (X.W.); wx893710305@163.com (X.W.); Liangjj19960926@163.com (J.-J.L.); ftt0809@163.com (T.-T.F.); zly1964@163.com (L.-Y.Z.); hxxxyyy1996@163.com (X.-Y.H.); 2 College of Chinese Materia Medica, Yunnan University of Chinese Medicine, Kunming 650500, China; guorui623974861@163.com; 3 School of Basic Medical Sciences, Guizhou University of Traditional Chinese Medicine, Guiyang 550025, China; 4 Department of Zoology & Yunnan Key Laboratory of Pharmacology for Natural Products, School of Pharmaceutical Sciences, Kunming Medical University, Kunming 650500, China; yufei5322032@163.com (H.-F.Y.); dingcaifeng@kmmu.edu.cn (C.-F.D.)

**Keywords:** gelselegandines D and E, indole alkaloids, osteoclast cells, *Gelsemium elegans*, osteoclastogenesis, natural products

## Abstract

The well-known toxic medicine *Gelsemium elegans* is widely and historically used to treat bone fracture and skin ulcers by the folk people of China. Two new monoterpenoid indole alkaloids, gelselegandines D and E, together with the known analogue gelegamine A were isolated from *G. elegans*. Their structures were elucidated by means of spectroscopic techniques and quantum chemical calculations. All isolated compounds were tested for the effects on RANKL-induced osteoclast formation. Interestingly, gelselegandine E and gelegamine A, respectively, showed significant promoting and inhibitory activities on osteoclastogenesis, while gelselegandine D had no activity under the same concentration. This work suggested the different configurations for the carbons near the C-19/20 oxygen rings of the isolated compounds may be the key active groups on osteoclast formation and provided the evidence for the rationality as the traditional treatment for bone-related diseases of *G. elegans*.

## 1. Introduction

Enlightened by the clinical practice of star natural products, pharmacists and chemists are always fascinated with their complicated structures as well as potent biological activities [1,2,3]. Previous studies intensively investigated the biological compounds and resulted in considerable groundbreaking discoveries [4,5,6]. With the aging of the population, osteoporosis (OP) caused by an imbalance of osteoclastic (OC) bone resorption became an increasing burden on global healthcare [7,8]. Targeting osteoclasts may serve as an effective treatment for osteolytic diseases [9]. In particular, due to the side effects of existing drugs, the market for osteoclasts’ formations’ regulators is scarce; it is urgent to screen safe and effective leading compounds applied in therapy for osteoporosis [9,10]. The search for structurally novel compounds regulating osteoclastogenesis has been a valuable topic in the fields of natural products, chemistry, biosynthesis, and organic synthesis.

**Figure 1:**
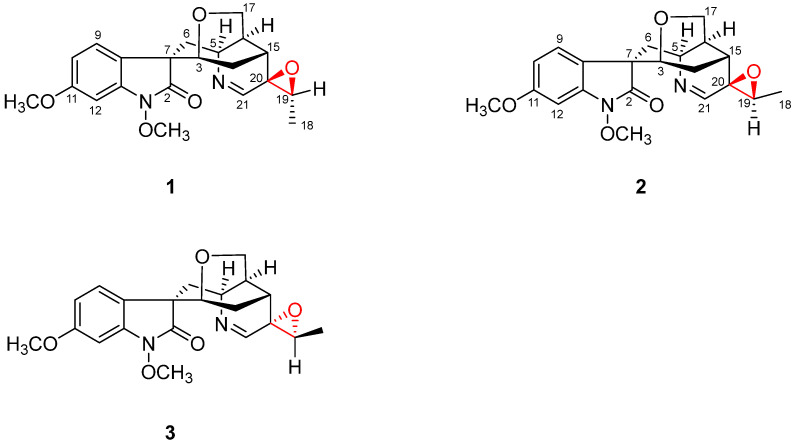
Structures of compounds **1–3**.

The well-known toxic medicine *Gelsemium elegans* belongs to the genus *Gelsemium*, called “Duan Chang Cao” or “Gou Wen” in the Traditional Chinese Medicine (TCM) [11,12,13]. *G. elegans* is widely and historically used to treat bone fracture and skin ulcers by the folk people of Guizhou province, China [14]. As one of the important plant sources for monoterpene indole alkaloids (MIAs), a series of novel indoles have been reported from different parts of *G. elegans* [12,13]. In our continuing research on MIAs medicine, gelselegandines D (**1**) and E (**2**), two new monoterpenoid indole alkaloids together with the known analogue gelegamine A (**3**) were isolated from the roots and stems of *G. elegans* (Figure 1). Structurally, compounds **1–3** are the humantenine-type alkaloids with a rare three-membered epoxy ring at C19/20. All isolated compounds were tested for the effects on the receptor activator for NF-κB ligand (RANKL)-induced osteoclast formation. Interestingly, compounds **2** and **3,** respectively, showed significant promoting and inhibitory activities on osteoclasts’ formation, while compound **1** had no activity under the same concentration, which suggested the different configurations for the carbons near the oxygen rings at C-19/20 of compounds **1–3** may be the key active groups on osteoclast formation. This work herein reports the extraction, isolation, and structural identification in detail, and provides the evidence for the rationality as the traditional treatment for bone-related diseases of *G. elegans*.

## 2. Results and Discussion

### 2.1. Structure Elucidation

Gelselegandine D (**1**), an amorphous solid, displayed a positive reaction to Dragendorff’s reagent. Its molecular formula was assigned as C_21_H_24_N_2_O_5_ by a quasi-molecular ion peak in HRESIMS at *m*/*z* 385.1751 [M + H]^+^ (calcd for C_21_H_25_N_2_O_5_^+^ 385.1758), corresponding to 10 degrees of unsaturation. The UV spectrum showed absorption maxima characteristic of an oxindole nucleus (215.5, 264.0 nm) [15]. The 1D NMR data (Table 1) showed signals ascribable to three methyl groups (*δ*_C_ 14.7, 56.2, 64.2), three methylenes (*δ*_C_ 37.1, 25.0, 67.9), nine methines including two oxygenated ones (*δ*_C_ 74.8, 60.7, 127.0, 109.3, 95.4, 31.3, 34.2, 64.6, 165.2), and six quaternary carbons (*δ*_C_ 174.4, 57.4, 125.1, 162.1, 140.6, 63.4). The above NMR data were closely related to those of gelegamine A (**3**) [15]. Additionally, the obvious chemical shifts’ difference in the carbons near epoxy three-membered rings of **1** and **3** suggested compound **1** may have different epoxy ring configurations from **3**.

**Figure 2:**
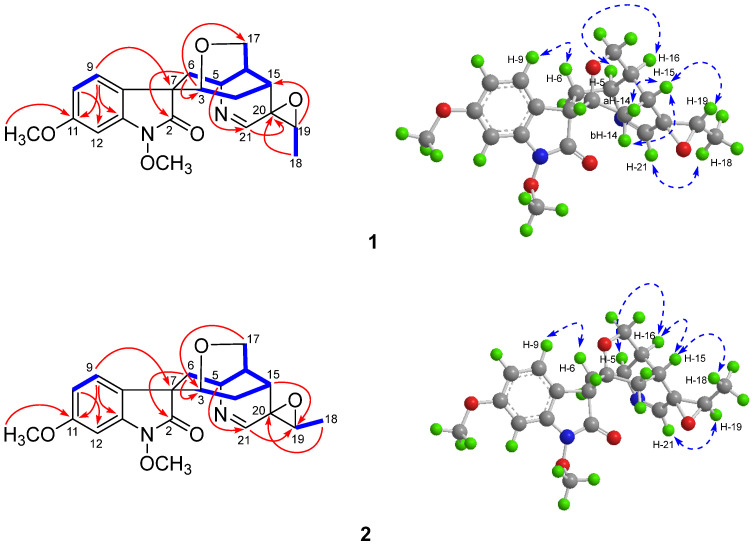
Selective HMBC (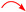), ^1^H-^1^H COSY (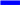), and ROESY (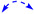) correlations of compounds **1** and **2**.

**Figure 3:**
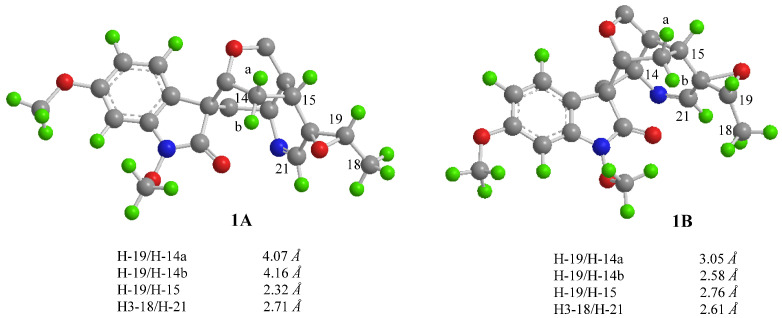
Calculated interproton distances near the epoxide for the two possible structures (**1A** and **1B**) of compound **1**.

In the rigid molecular model of humantenine-type alkaloids including compound **1**, the cage-like skeletons required the *β*-orientations for H-3 and H-15. In the ROESY spectrum of **1** (Figure 2), the cross peaks observed between the proton pairs H-6/H-9 and H-5/H-16, respectively, indicated the relative configuration of C-7, C-5, and C-16, which were identical to that of **3** [15]. Besides, the NOE correlations of H-15/H-19 and H-21/H-18 indicated the *cis*-configuration of the epoxy three-membered ring at C19/20. Thus, the relative configurations of **1** possessed only two possibilities, as shown in Figure 3, including the *β* (**1A**) and *α* (**1B**) orientations of the epoxy ring at C19/20 [15]. The distances of the proton pairs near the epoxy ring of the two optimized structures of **1A** and **1B** (Figure 3) were calculated [16], of which **1A** was completely consistent with the corresponding experimental ROESY data of **1**.

**Figure 4:**
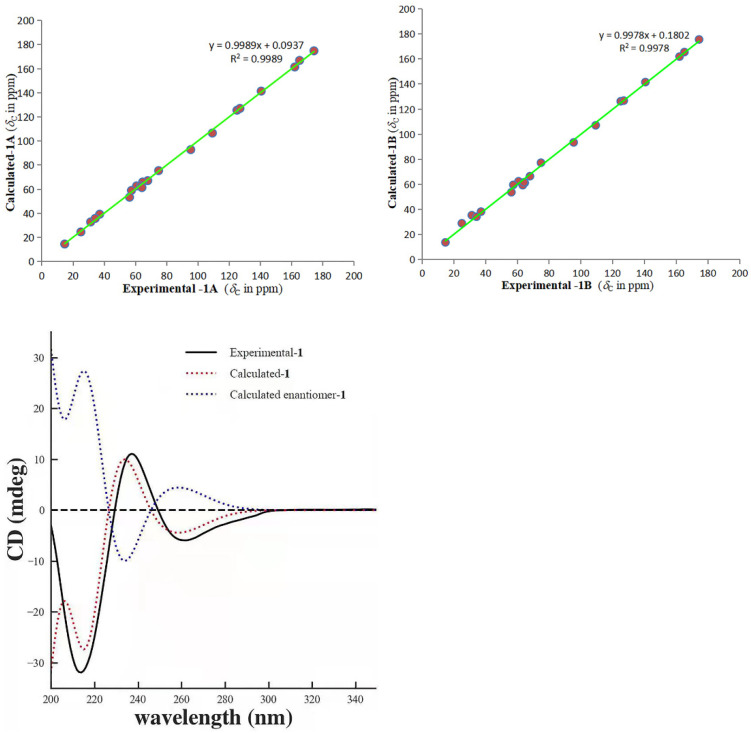
Regression analysis of the NMR calculations of the two possible configurations (**1A** and **1B**) and ECD calculation curves of **1**.

Furthermore, the ^13^C NMR calculations of the two possible conformers (**1A** and **1B**) were performed [11,17]. The result showed (Figure 4) the correlation coefficient (R^2^), corrected mean absolute deviation (CMAD), and corrected largest absolute deviation (CLAD) of the **1A** [R^2^ = 0.9989, CMAD = 1.34 ppm, CLAD = 2.93 ppm] were better than that of **1B** [R^2^ = 0.9978, CMAD = 1.84 ppm, CLAD = 4.08 ppm] (Supplementary Materials), which established the *β* (**1A**) orientation of the epoxy ring at C19/20 of **1**. Meanwhile, the Electronic circular dichroism (ECD) calculation [11] was used to determine the absolute configuration of **1**, and the calculated ECD curve for 3*R*, 5*S*, 7*S*, 15*R*, 16*S*, 19*S*, and 20*R*-**1** was in good agreement with the experimental values of **1.** Therefore, compound **1** was determined to be gelselegandine D, as shown in Figure 1.

**Figure 5:**
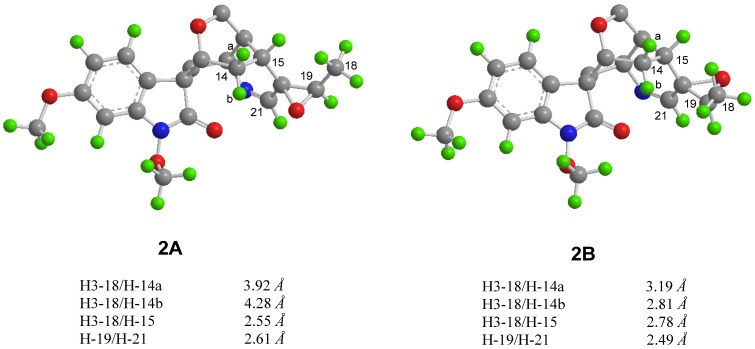
Calculated interproton distances near the epoxide for the two possible structures (**2A** and **2B**) of compound **2**.

**Figure 6:**
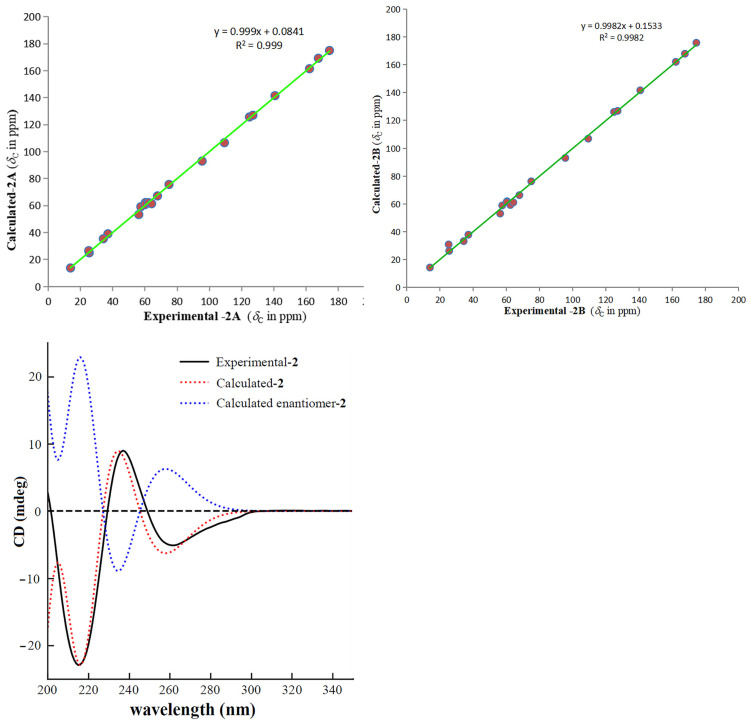
Regression analysis of the NMR calculations of the two possible configurations (**2A** and **2B**) and ECD calculation curves of **2**.

Gelselegandine E (**2**), an amorphous solid, displayed a positive reaction to Dragendorff’s reagent. The molecular formula of **2** was established as C_21_H_24_N_2_O_5_ (10 degrees of unsaturation) from its HRESIMS (*m*/*z* 385.1752 [M + H] ^+^, calcd for C_21_H_25_N_2_O_5_^+^: 385.1758). The 1D and 2D NMR spectra of **2** (Table 1) were similar to those of **1**, except that the *cis*-configuration of the epoxy ring at C19/20 in **1** was replaced by a *trans*-configuration of the epoxy ring at C19/20 in **2**, which was suggested by the NOE correlations of H-15/H-18 and H-21/H-19 in the ROESY spectrum of **2**. Consistent with compound **1**, the relative configuration of compound **2** also possessed two possibilities, as shown in Figure 5, including the *β* (**2A**) and *α* (**2B**) orientations of the epoxy ring at C19/20 [15]. The calculated distances of the proton pairs near the epoxy ring of the optimized structures of **2A** (Figure 5) were completely consistent with the corresponding experimental ROESY data of **2** [16]. Besides, the ^13^C NMR calculations showed (Figure 6) the correlation coefficient (R^2^), corrected mean absolute deviation (CMAD), and corrected largest absolute deviation (CLAD) of the **2A** [R^2^ = 0.9990, CMAD = 1.25 ppm, CLAD = 2.86 ppm] were better than that of **2B** [R^2^ = 0.9982, CMAD = 1.62 ppm, CLAD = 5.69 ppm] (Supplementary Materials), which established the *β* (**2A**) orientation of the epoxy ring at C19/20 of **2** [17]. The ECD calculations finally determined (Figure 6) the absolute configuration of **2** as 3*R*, 5*S*, 7*S*, 15*R*, 16*S*, 19*R*, and 20*R,* as shown in Figure 1 [11].

In addition to new compounds **1** and **2**, one known analogue, gelegamine A (**3**), was also isolated and identified by comparison with reported data [15].

### 2.2. The Effects of Compounds **1**–**3** on Osteoclastogenesis Induced by RANKL

**Figure 7:**
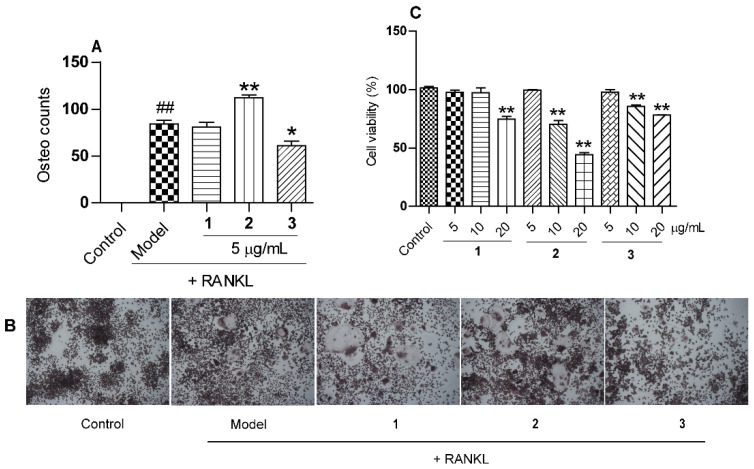
Effects of compounds **1–3** on RANKL-induced osteoclast formation; (**A**) osteoclast counts after compounds’ addition (^##^ *p* < 0.01 versus control; * *p* < 0.05, ** *p* < 0.01 versus model); (**B**) representative TRAP staining images after compounds’ intervention; (**C**) dell viability of the compounds with various concentrations (5, 10, or 20 μg/mL) on macrophages RAW 264.7 (** *p* < 0.01 *versus* control). RANKL, receptor activator for NF-κB ligand; TRAP, tartrate-resistant acid phosphatase; Osteo, osteoclast.

The cytotoxicity of the isolated compounds **1–3** on RAW 264.7 cells was measured using the MTT assay. The results showed that all the compounds (**1–3**) exhibited no cytotoxicity at the concentration of 5 μg/mL (Figure 7). The receptor activator for NF-κB ligand (RANKL)-induced osteoclast from macrophages RAW 264.7 were widely used to evaluate the effect of treatments on osteoclast formation [18]. The effects of compounds **1–3** on osteoclastogenesis assay induced by RANKL were tested. As shown in Figure 7A,B, compounds **2** and **3** (5 μg/mL), respectively, promoted and inhibited the formation of osteoclasts significantly after RANKL addition, while compound **1** was not active under the same concentration, which suggested the different configurations for the carbons near the C-19/20 oxygen rings of compounds **1–3** may be the key factor on osteoclastogenesis.

## 3. Experimental Section

### 3.1. General Experimental Procedures

Optical rotation was performed on an Autopol VI, Serial #91058. IR spectra were measured on a NICOLET iS10 spectrometer with KBr pellets. UV spectra were obtained on Agilent 8453. 1D and 2D NMR spectra were recorded on a Bruker Avance NEO (600 MHz). Coupling constants are expressed in Hertz, and chemical shifts are given on a ppm scale with tetramethylsilane as an internal standard. HRESIMS was recorded on a Thermo Fisher QE Focus spectrometer. CD spectra were obtained on a JASCO 810 spectrometer. Column chromatography (CC) was performed on silica gel (200–300 mesh, Qingdao Marine Chemical Ltd., Qingdao, China), GE Sephadex LH-20 (GE Healthcare Bio-Sciences, Uppsala, Sweden), and MCI-gel (Mitsubishi Chemical Co., Ltd., Tokyo, Japan). Thin-layer chromatography (TLC) was carried out on silica gel GF-254 precoated plates, which were purchased from Qingdao Haiyang Chemical Co., Ltd., with CH_2_Cl_2_/MeOH (9:1, 4:1, *v*/*v*) as developing solvents. High-performance liquid chromatography (HPLC) was performed on SEP LC-52 with an MWD UV detector (Separation Technology Co., Ltd., Beijing, China) and preparative C_18_ columns (250 × 20 mm). Staining kits for the 3-(4,5-Dimethylthiazol-2-yl)-2,5-diphenyltetrazolium bromide (MTT) assay and tartrate-resistant acid phosphatase (TRAP) were obtained from Sigma-Aldrich (Saint Louis, MO, USA). *Escherichia coli*-derived recombinant mouse RANKL was purchased from R&D Systems (Minneapolis, MN, USA).

### 3.2. Plant Material

The roots and stems of *G. elegans* were purchased from Kunming Qian Cao Yuan herbal medicine shop, Yunnan province, China, in November 2019, and identified by Professor Sheng-Hua Wei, from Guizhou University of Traditional Chinese Medicine. A voucher specimen (No. WX_20191101) was deposited in Guizhou University of Traditional Chinese Medicine.

### 3.3. Extraction and Isolation

The air-dried and powdered roots and stems of *G. elegans* (5 kg) were extracted with MeOH (20 L × 3) under reflux conditions at 70 °C, 3 h for each time. After the removal of the organic solvent under reduced pressure, a crude extract (311.04 g) was obtained, and the HCl solution (pH 2) was added with stirring to dissolve and filter the extract. The filter liquor was adjusted to pH 10 with ammonium hydroxide, then further extracted with EtOAc (5 L × 4) to give an extract. The extract (30 g) was subjected to a silica gel column (CH_2_Cl_2_/MeOH, 1:0–0:1) to afford fractions (A-F). Fr. D (9.67 g) was subjected to a silica gel column (CH_2_Cl_2_: EtOAc, 18:1–0:1; CH_2_Cl_2_: MeOH, 16:1–0:1) to afford 12 fractions (Fr.D1-D12). Fr.D3 (757.40 mg) was further subjected to Sephadex LH-20 column chromatography (CC) using MeOH under isocratic conditions to give a mixture (570.3 mg), which was then further separated on a MCI-gel CHP 20P column with a gradient of MeOH/H_2_O (3:7–1:0) and preparative C_18_ HPLC column under a gradient of MeOH/H_2_O (3:2–1:0) to get **1** (4.30 mg), **2** (6.08 mg), and **3** (2.41 mg).

#### 3.3.1. Gelselegandine D (**1**)

Amorphous solid; [α]^2^^1^_D_ -295.83 (*c* 0.097, MeOH); UV (MeOH) *λ*_max_: 284.0, 264.0, and 215.5 nm; IR (KBr) *ν*_max_ 3429, 2932, 1721, 1628, 1496, 1216, 1113, 1039, and 994 cm^−1^; HRESIMS *m*/*z* 385.1751 [M + H]^+^ (calcd for C_21_H_25_N_2_O_5_^+^ 385.1758); ^1^H and ^13^C NMR data, see Table 1.

#### 3.3.2. Gelselegandine E (**2**)

Amorphous solid; [α]^21^_D_ -254.09 (*c* 0.132, MeOH); UV (MeOH) *λ*_max_: 284.5, 268.5, and 216.5 nm; IR (KBr) *ν*_max_ 3434, 2936, 1723, 1629, 1497, 1216, 1114, 1038, and 993 cm^−1^; HRESIMS *m*/*z* 385.1752 [M + H]^+^ (calcd for C_21_H_25_N_2_O_5_^+^ 385.1758); ^1^H and ^13^C NMR data, see Table 1.

### 3.4. ECD Calculation

The ECD calculations of **1** and **2** were carried out using previous means [11]. The detailed description of this section is provided in Supplementary Materials.

### 3.5. ^13^C NMR Calculation

NMR calculations were carried out following the protocol reported in [11], and the description of the experimental process is provided in Supplementary Materials.

### 3.6. Cell Culture and Cell Viability Assay

A murine macrophage cells’ line (RAW 264.7) was obtained from American Type Culture Collection (Manassas, VA, USA). Cells were incubated in Dulbecco’s modified Eagle’s medium (DMEM) supplemented with 10% fetal bovine serum (FBS), penicillin (100 U/mL), and streptomycin (100 μg/mL) at 37 °C in an atmosphere of 5% CO_2_.

The cell viability of RAW 264.7 cells was measured after compounds’ treatment using the MTT assay. Briefly, cells were seeded on 96-well plates at a density of 2 × 10^4^/well and incubated overnight. Cells were exposed to compounds **1–3** (5, 10, or 20 μg/mL) for an additional 72 h. After incubation for a specified time, 20 μL of MTT were added into each well and incubation was allowed for an additional 4 h. Then, cells’ supernatants were removed, followed by the addition of 200 μL of dimethyl sulfoxide to dissolve formazan crystals. Absorbance was measured at 492 nm with a FlexStation^TM^ 3 Multi-Mode Microplate Reader (Molecular Devices, Silicon Valley, CA, USA). Results are expressed as a percentage of the control.

### 3.7. Osteoclastogenesis Assay In Vitro

The 96-well plates were used to culture RAW 264.7 cells at 2 × 10^3^ cells/well overnight. The following day, compounds **1–3** (5 μg/mL) were used to pretreat cells for 30 min, and then cells were stimulated with RANKL (50 ng/mL) for another 5 days. Then, plates were fixed with fixative solution by combining Citrate Solution, acetone, and 37% formaldehyde and staining with TRAP, according to manufacturer’s instructions. TRAP-positive cells that contained >3 nuclei were regarded as mature osteoclasts and counted using a light microscope.

## 4. Conclusions

In summary, two new monoterpenoid indole alkaloids, gelselegandines D and E, together with the known analogue gelegamine A were isolated from *G. elegans.* Their structures were elucidated by means of spectroscopic techniques and quantum chemical calculations. Structurally, the isolated compounds are the humantenine-type alkaloids with a rare, three-membered epoxy ring at C19/20. Further, RANKL-induced osteoclast formation bioassays were carried out. It is worth mentioning that gelselegandine E and gelegamine A, respectively, showed significant promoting and inhibitory activities on osteoclasts formation, while gelselegandine D had no activity under the same concentration, which suggested the different configurations for the carbons near the oxygen rings at C-19/20 of the tested compounds may be the key active groups on osteoclast formation. This work provided the evidence for the rationality as the traditional treatment for bone-related diseases of *G. elegans*.

## Figures and Tables

**Table 1:** The ^1^ H (600 MHz) and ^13^ C NMR (150 MHz) data for **1**–**2** (*δ* in ppm, *J* in Hz).

NO.	1 ^a^		2 ^a^	
	*δ*_H_	*δ*_C_	*δ*_H_	*δ*_C_
2		174.4		174.5
3	3.57 (d, *J* = 8.2, 1H),	74.8	3.63 (d, *J* = 7.8, 1H)	74.9
5	4.32 (tt, *J* = 7.3, 2.1, 1H)	60.7	4.26 (tt, *J* = 7.2, 2.4, 1H)	60.4
6	2.66 (dd, *J* = 15.8, 7.2, 1H), 2.21 (d, *J* = 15.8, 1H)	37.1	2.61 (dd, *J* = 15.7, 7.2, 1H), 2.14 (dd, *J* = 15.7, 2.2, 1H)	37.1
7		57.4		57.4
8		125.1		124.8
9	7.39 (d, *J* = 8.3, 1H)	127.0	7.40 (d, *J* = 8.3, 1H)	127.0
10	6.66 (dd, *J* = 8.3, 2.4, 1H)	109.3	6.66 (dd, *J* = 8.3, 2.4, 1H)	109.3
11		162.1		162.1
12	6.58 (d, *J* = 2.4, 1H)	95.4	6.58 (d, *J* = 2.4, 1H)	95.5
13		140.6		140.6
14	2.55 (m, 1H), 2.05 (ddd, *J* = 15.3, 11.2, 8.3, 1H)	25.0	2.57 (dd, *J* = 15.2, 5.4, 1H), 2.07 (ddd, *J* = 15.2, 11.5, 7.9, 1H)	25.5
15	1.91 (m, 1H)	31.3	2.18 (m, 1H)	25.1
16	2.56 (m, 1H)	34.2	2.44 (m, 1H)	34.2
17	4.41 (d, *J* = 10.5, 1H), 4.03 (dd, *J* = 10.5, 4.0, 1H)	67.9	4.40 (d, *J* = 10.5, 1H), 4.06 (dd, *J* = 10.5, 3.9, 1H)	67.8
18	1.45 (d, *J* = 5.6, 3H)	14.7	1.40 (d, *J* = 5.4, 3H)	13.9
19	3.24 (q, *J* = 5.6, 1H)	64.6	3.33 (m, 1H)	60.1
20		63.4		62.3
21	7.61 (s, 1H)	165.2	7.25 (s, 1H)	167.6
NOMe	3.93 (s, 3H)	64.2	3.93 (s, 3H)	64.2
ArOMe	3.82 (s, 3H)	56.2	3.82 (s, 3H)	56.2

^a^ Recorded in CD_3_OD.

## Data Availability

Not applicable.

## Supplementary Materials

The following are available online. The detailed ECD and NMR calculation, 1D and 2D NMR spectra, HRESIMS, UV, IR, and ECD spectra of compounds **1** and **2** [19,20,21].

## Sample Availability

Samples of the compounds are not available from the authors.
