# Supplementary material for: New Monoterpenoid Indoles with Osteoclast Activities from Gelsemium elegans
Source: Molecules. 2021 Dec 9;26(24):7457. doi: 10.3390/molecules26247457 (PMC8708120; doi:10.3390/molecules26247457)
Supplement: Supplementary file 1 [file molecules-26-07457-s001.zip › molecules-1496620-supplementary.pdf]

molecules

Supplementary Materials

New Monoterpenoid Indoles with Osteoclast Activities from *Gelsemium elegans*

Xin Wei [1,†], Rui Guo [2,†], Xiao Wang [1,3,†], Jia-Jun Liang [1], Hao-Fei Yu [4], Cai-Feng Ding [4], Ting-Ting Feng [1], Li-Yan Zhang [1], Xia Liu [1,3,*], Xin-Yue Hu [1] and Ying Zhou [1,*]

[1] School of Pharmacy, Guizhou University of Traditional Chinese Medicine, Guiyang 550025, China; sfweixin@163.com (X.W.); wx893710305@163.com (X.W.); Liangjj19960926@163.com (J.-J.L.); ftt0809@163.com (T.-T.F.); zly1964@163.com (L.-Y.Z.); hxxxyyy1996@163.com (X.-Y.H.)
[2] College of Chinese Materia Medica, Yunnan University of Chinese Medicine, Kunming 650500, China; guorui623974861@163.com
[3] School of Basic Medical Sciences, Guizhou University of Traditional Chinese Medicine, Guiyang 550025, China
[4] Department of Zoology & Yunnan Key Laboratory of Pharmacology for Natural Products, School of Pharmaceutical Sciences, Kunming Medical University, Kunming 650500, China; yufei5322032@163.com (H.-F.Y.); dingcaifeng@kmmu.edu.cn (C.-F.D.)
* Correspondence: liuxia0851@126.com (X.L.); yingzhou71@sina.com (Y.Z.)
† These authors contributed equally to this work.

CONTENTS

1. The spectra of compound 1

Figure S1. ^{1}H NMR spectrum of compound **1**.
Figure S2. ^{13}C NMR spectrum of compound **1**.
Figure S3. HSQC spectrum of compound **1**.
Figure S4. HMBC spectrum of compound **1**.
Figure S5. ^{1}H-^{1}H COSY spectrum of compound **1**.
Figure S6. ROESY spectrum of compound **1**.
Figure S7. ECD spectrum of compound **1**.
Figure S8. HRESIMS spectrum of compound **1**.
Figure S9. UV spectrum of compound **1**.
Figure S10. IR spectrum of **1**.

2. The spectra of compound 2

Figure S11. ^{1}H NMR spectrum of compound **2**.
Figure S12. ^{13}C NMR spectrum of compound **2**.
Figure S13. HSQC spectrum of compound **2**.
Figure S14. HMBC spectrum of compound **2**.
Figure S15. ^{1}H-^{1}H COSY spectrum of compound **2**.
Figure S16. ROESY spectrum of compound **2**.
Figure S17. ECD spectrum of compound **2**.
Figure S18. HRESIMS spectrum of compound **2**.
Figure S19. UV spectrum of compound **2**.
Figure S20. IR spectrum of **2**.

3. ECD and ^{13}C NMR calculations of compound 1

4. ECD and ^{13}C NMR calculations of compound 2

References

1. The spectra of compound 1

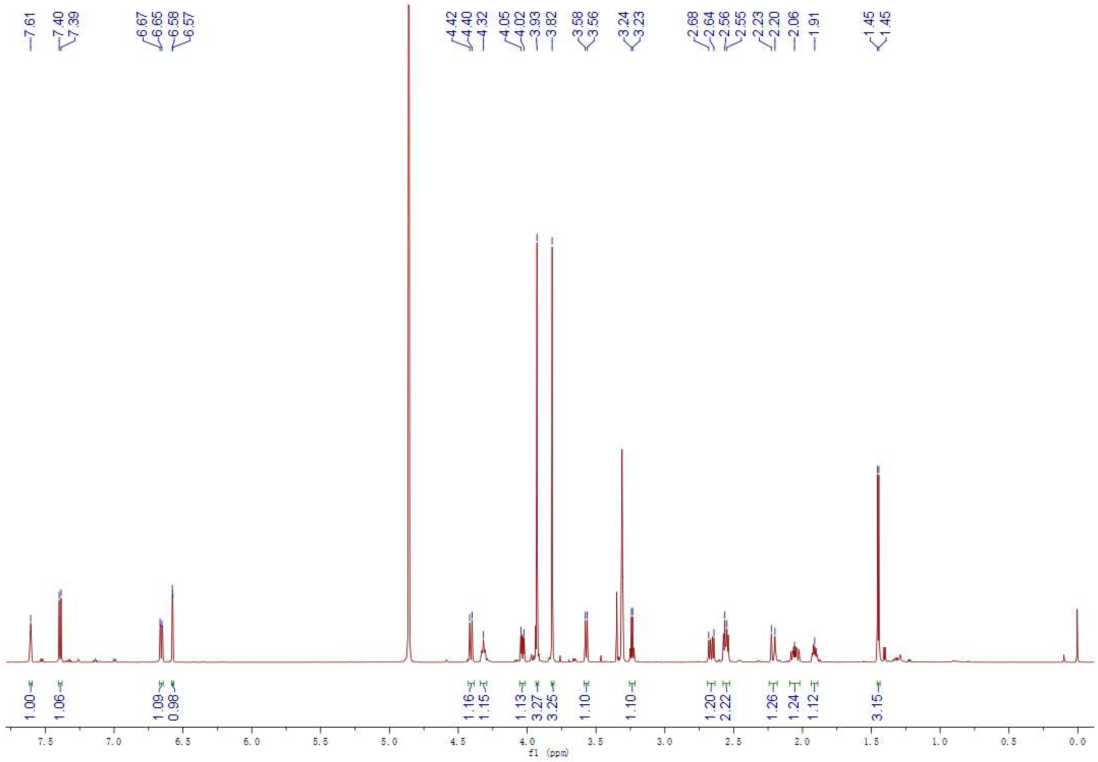

Figure S1. ^{1}H NMR spectrum of compound **1.**

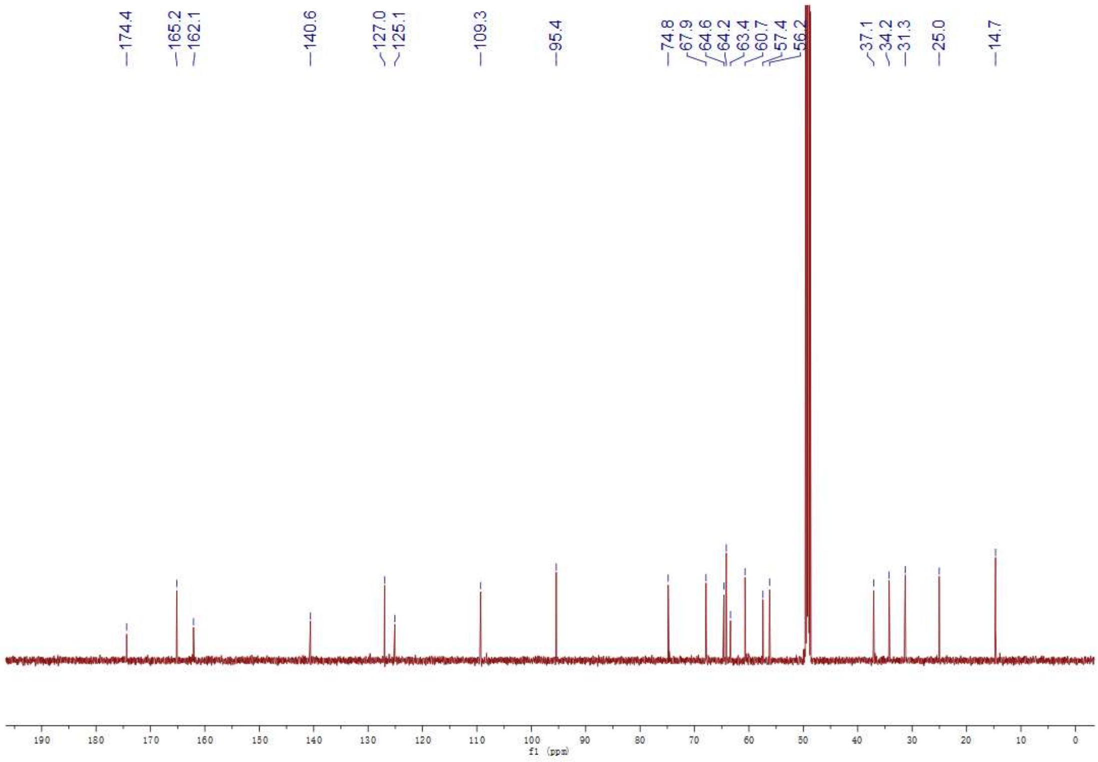

Figure S2. ^{13}C NMR spectrum of compound **1.**

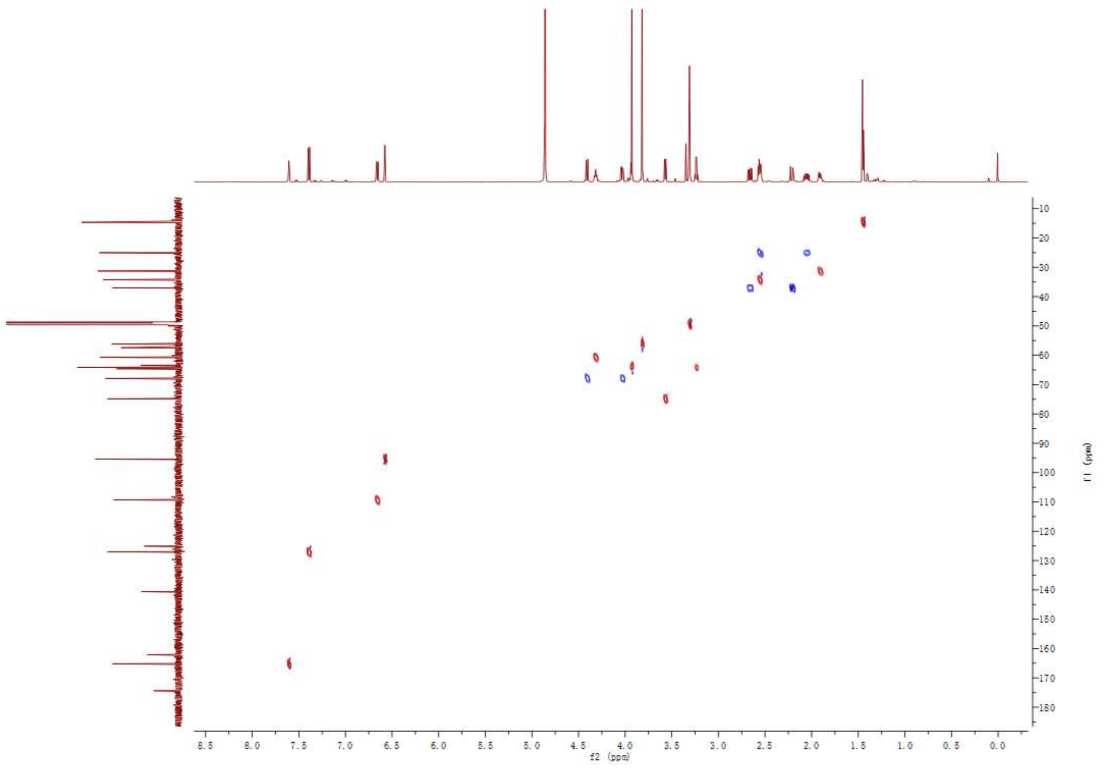

Figure S3. HSQC spectrum of compound **1.**

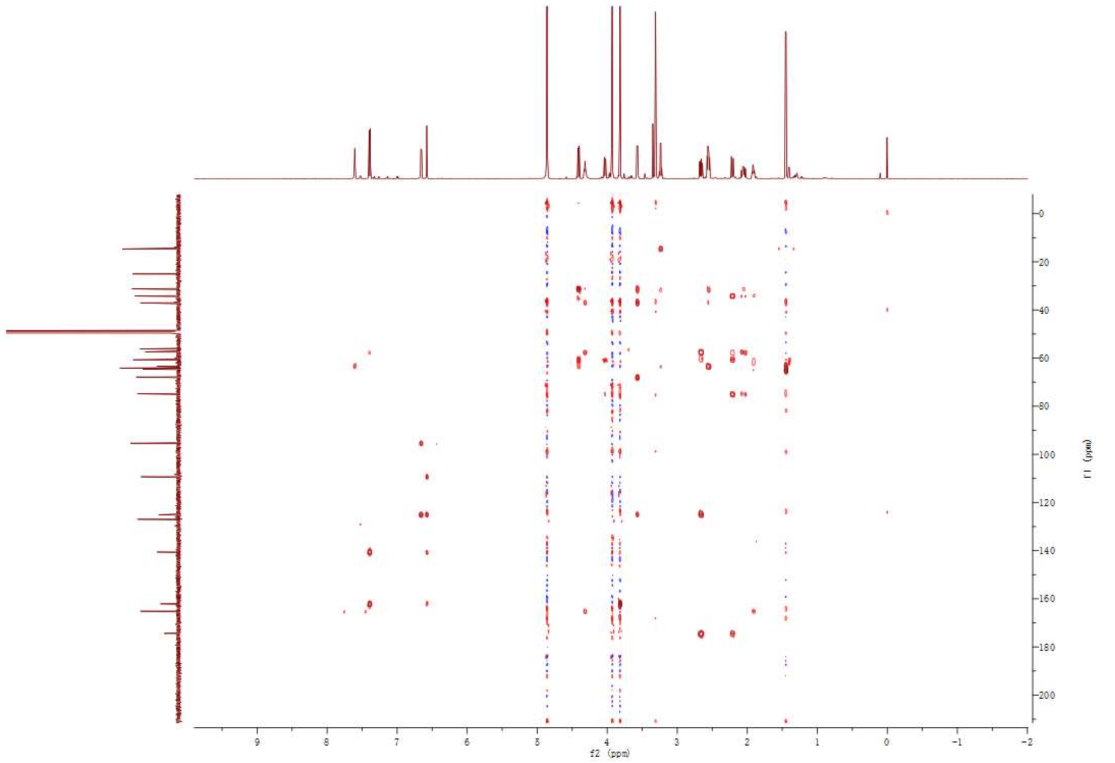

Figure S4. HMBC spectrum of compound **1.**

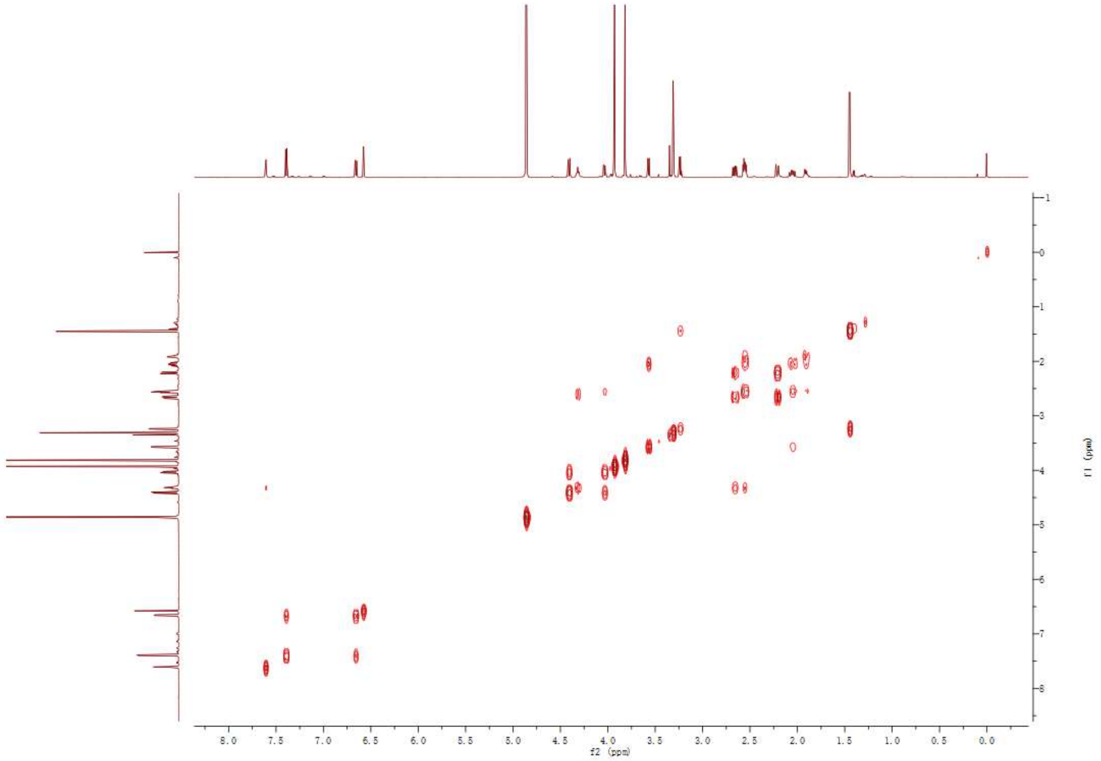

Figure S5. ^{1}H-^{1}H COSY spectrum of compound **1.**

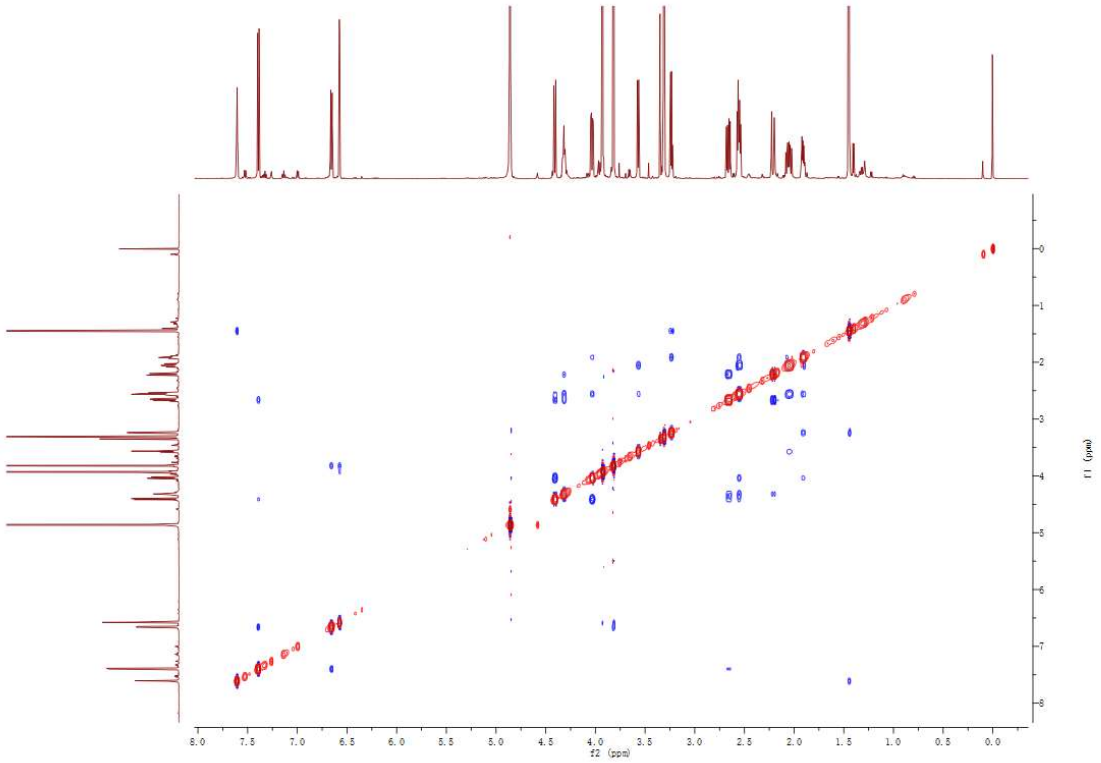

Figure S6. ROESY spectrum of compound **1.**

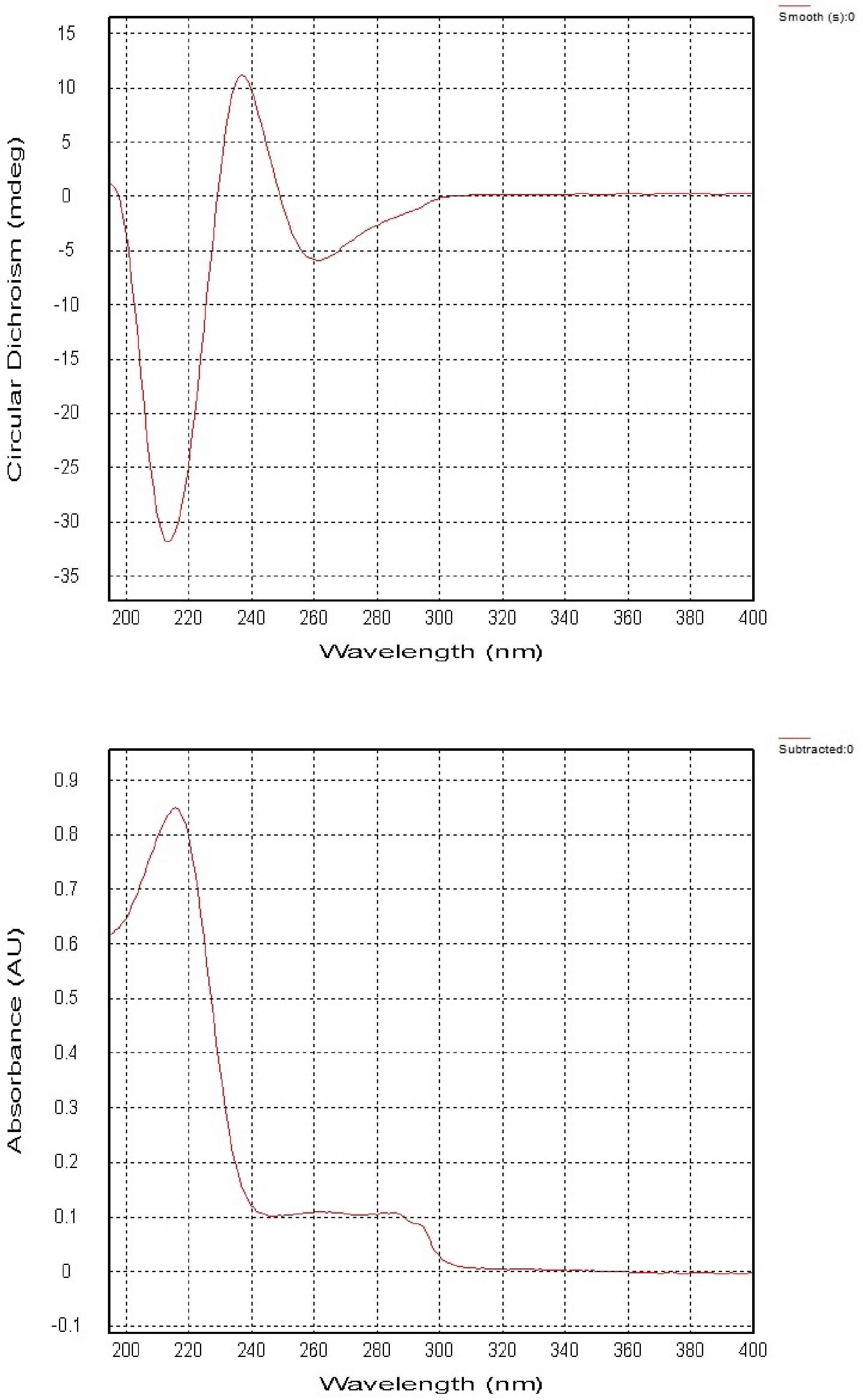

Figure S7. ECD spectrum of compound **1.**

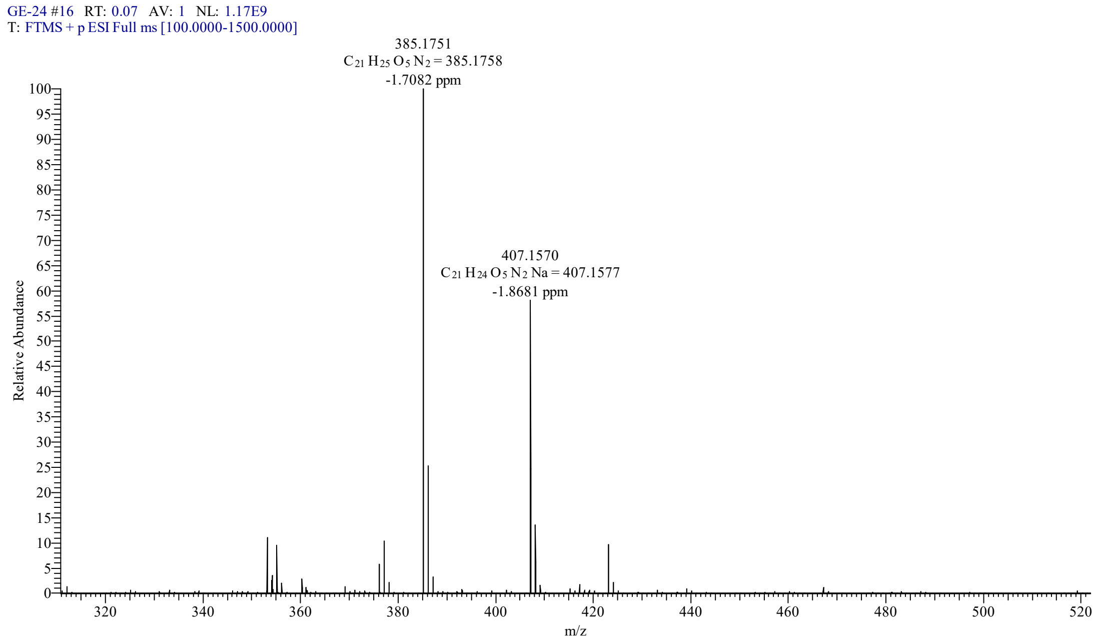

Figure S8. HRESIMS spectrum of compound **1.**

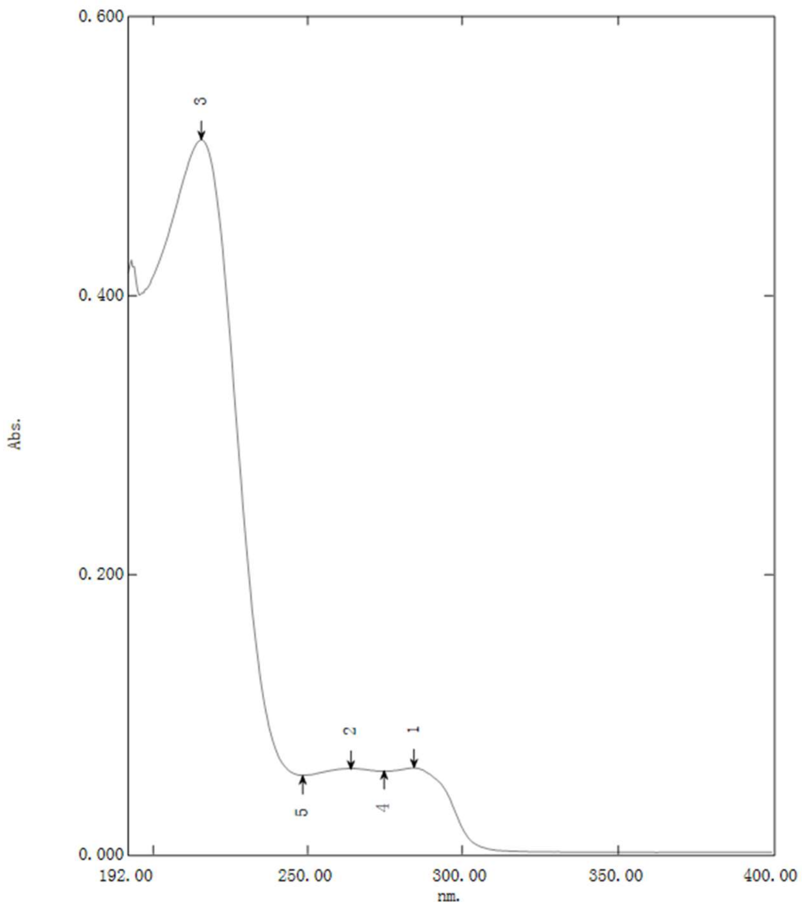

No.	P/V	波长(nm)	Abs.	描述
1	↑	284. 00	0. 062	
2	↑	264. 00	0. 061	
3	↑	215. 50	0. 512	
4	↓	274. 50	0. 059	
5	↓	248. 50	0. 056	

Figure S9. UV spectrum of compound **1.**

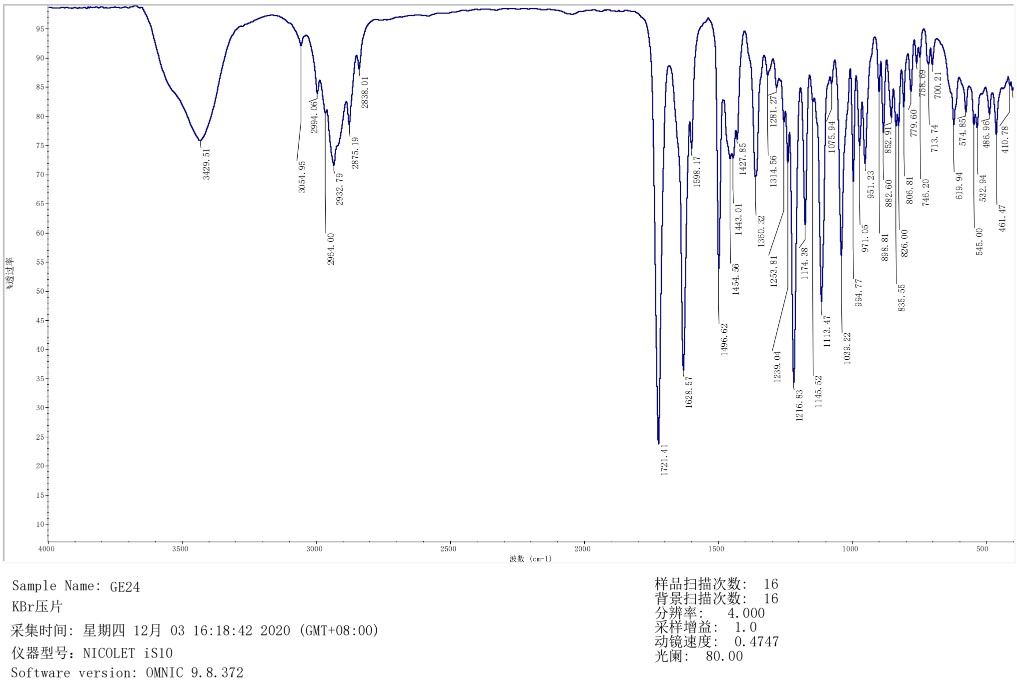

Figure S10. IR spectrum of 1.

2. The spectra of compound 2

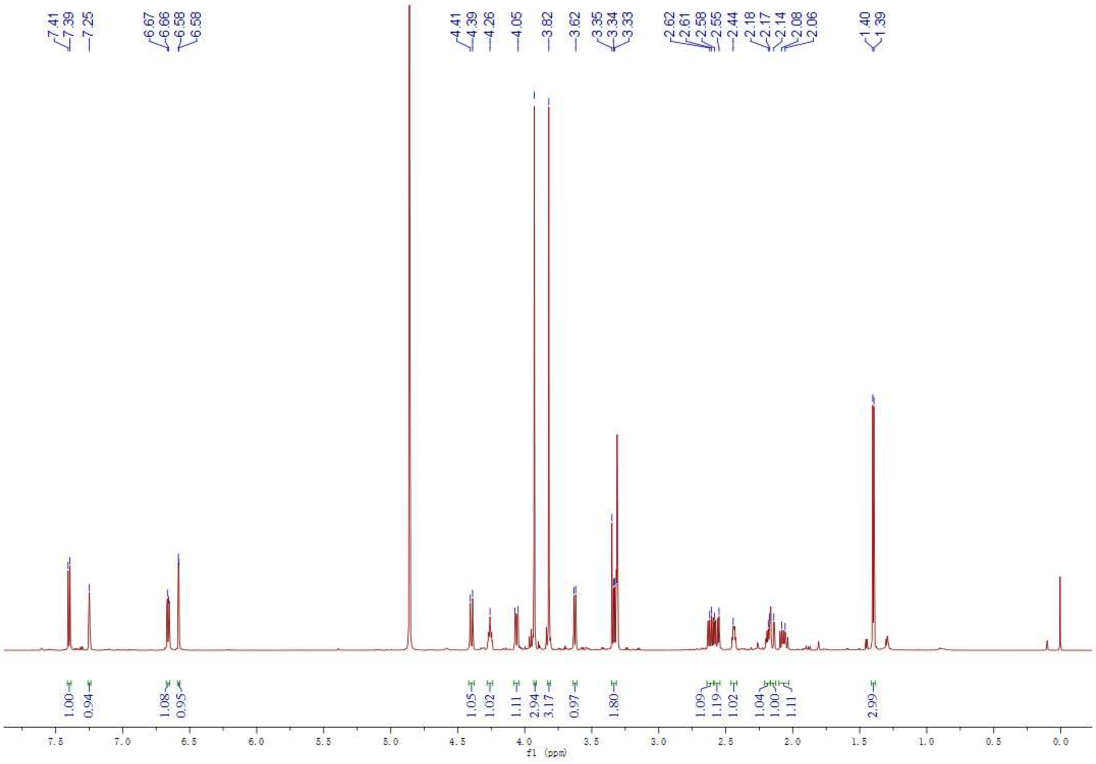

Figure S11. ^{1}H NMR spectrum of compound **2**.

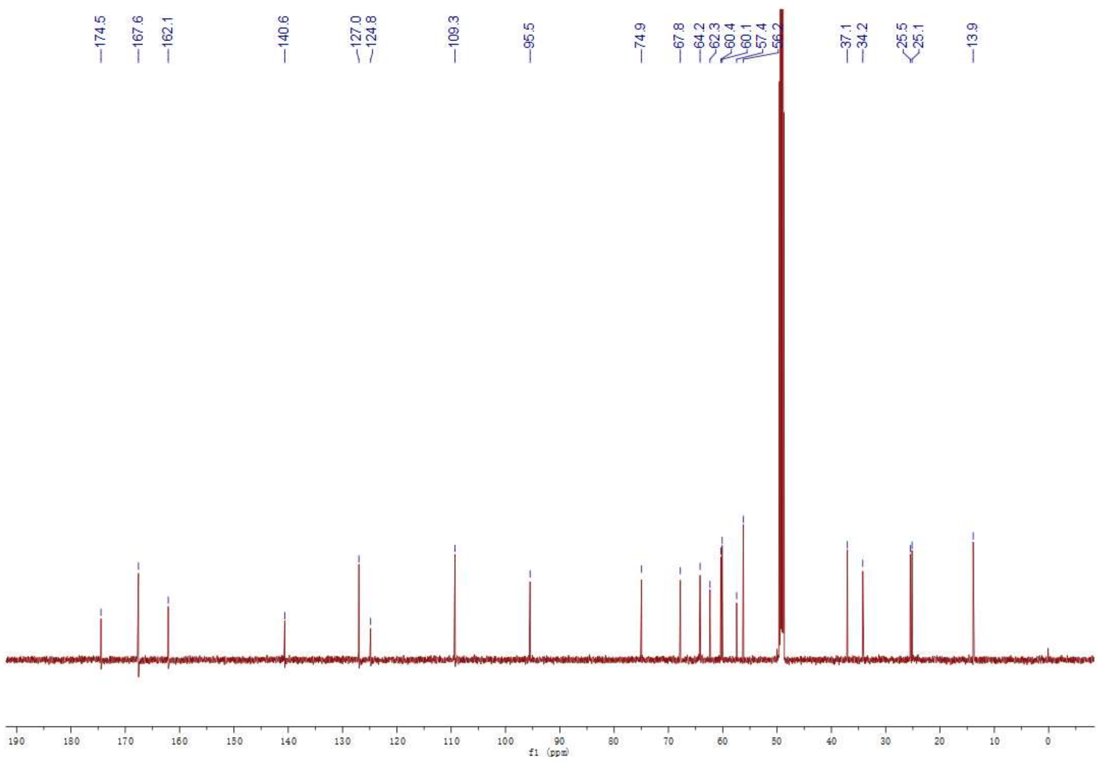

Figure S12. ^{13}C NMR spectrum of compound **2**.

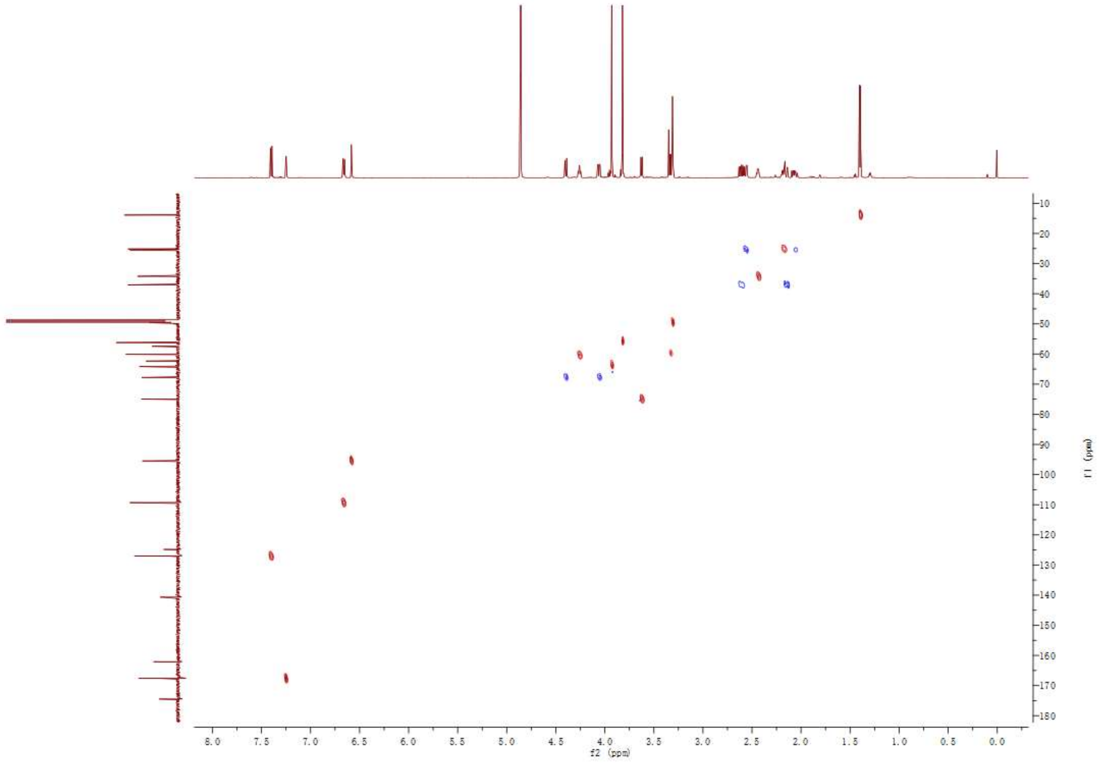

Figure S13. HSQC spectrum of compound **2**.

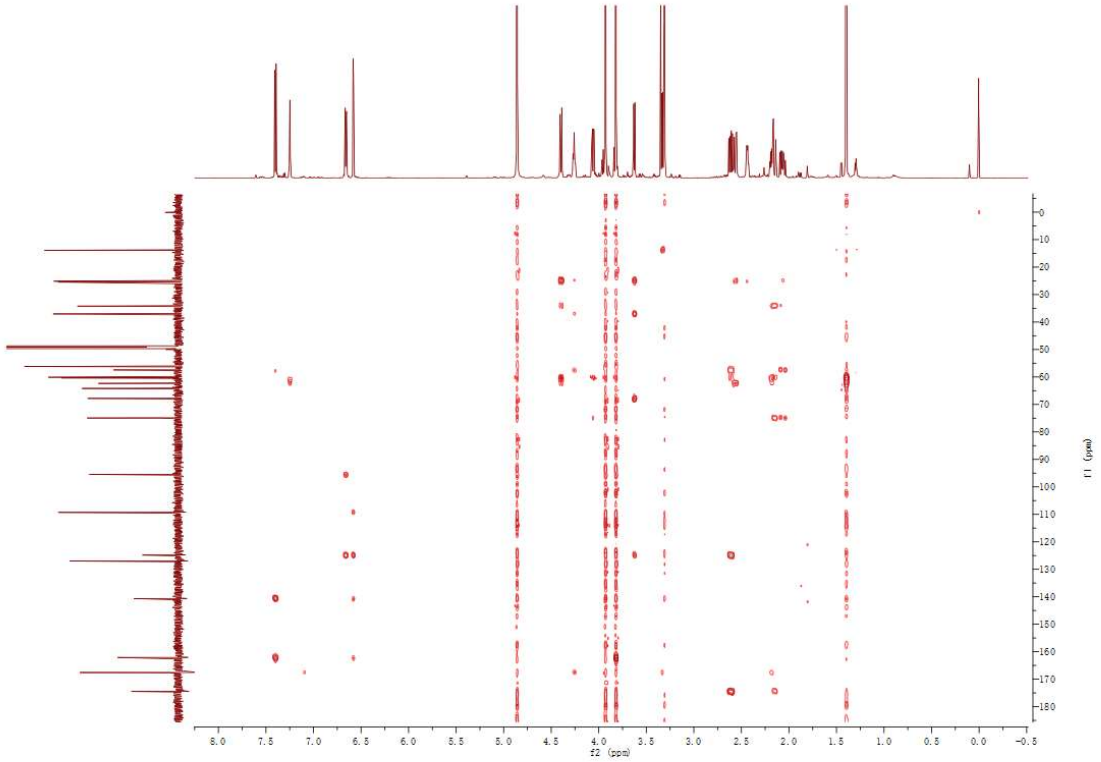

Figure S14. HMBC spectrum of compound **2**.

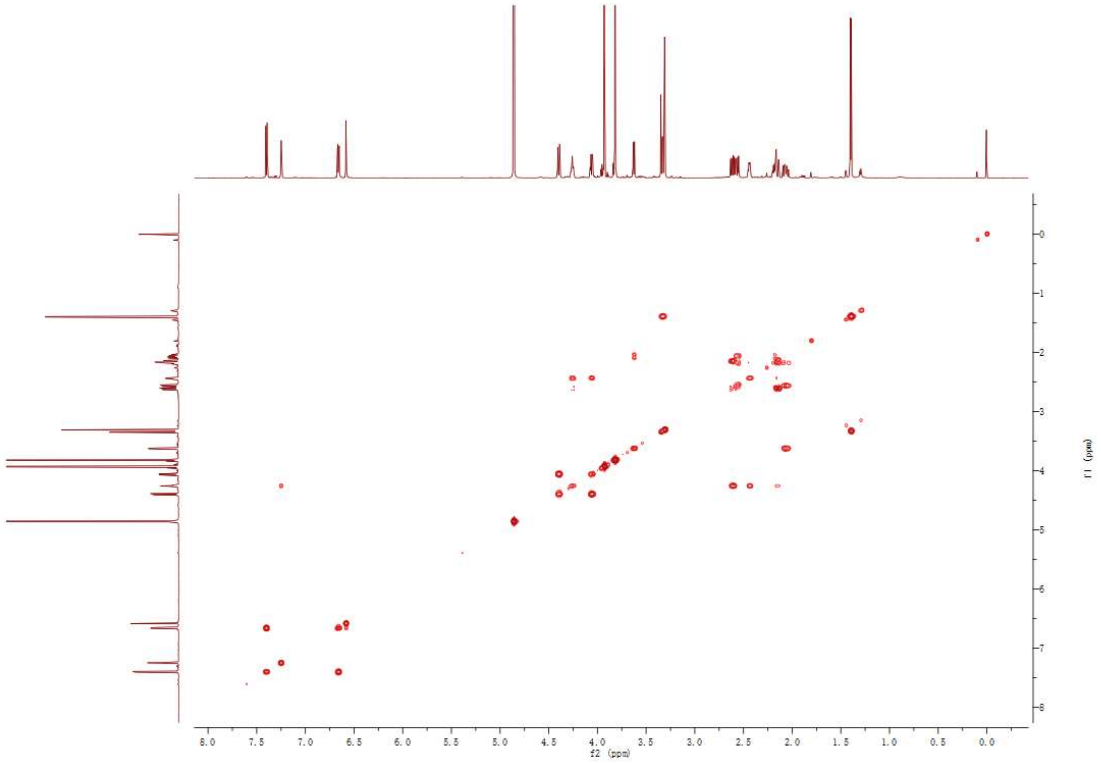

Figure S15. ^1H-^1H COSY spectrum of compound **2**.

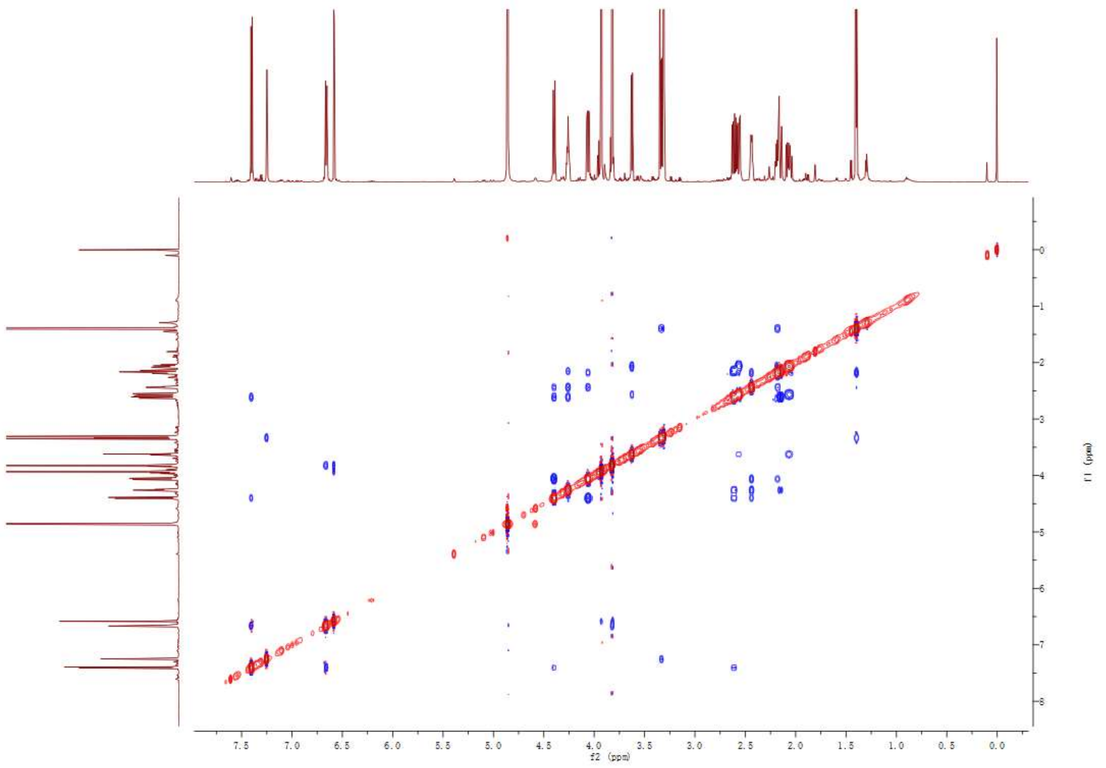

Figure S16. ROESY spectrum of compound **2**.

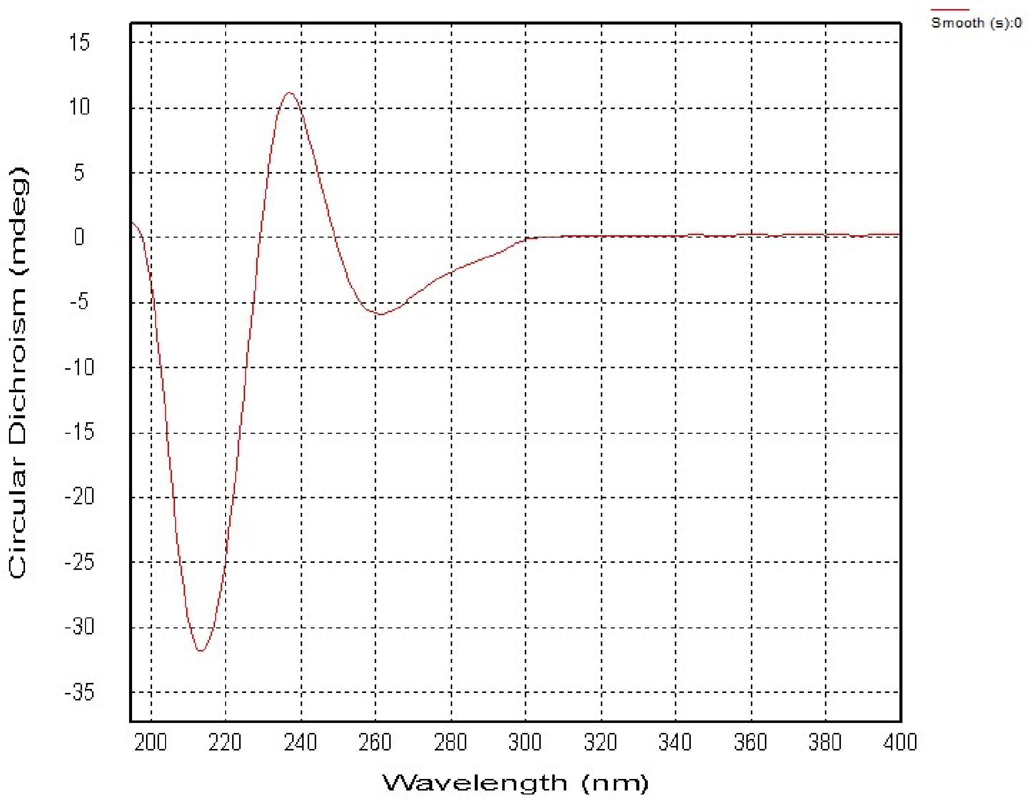

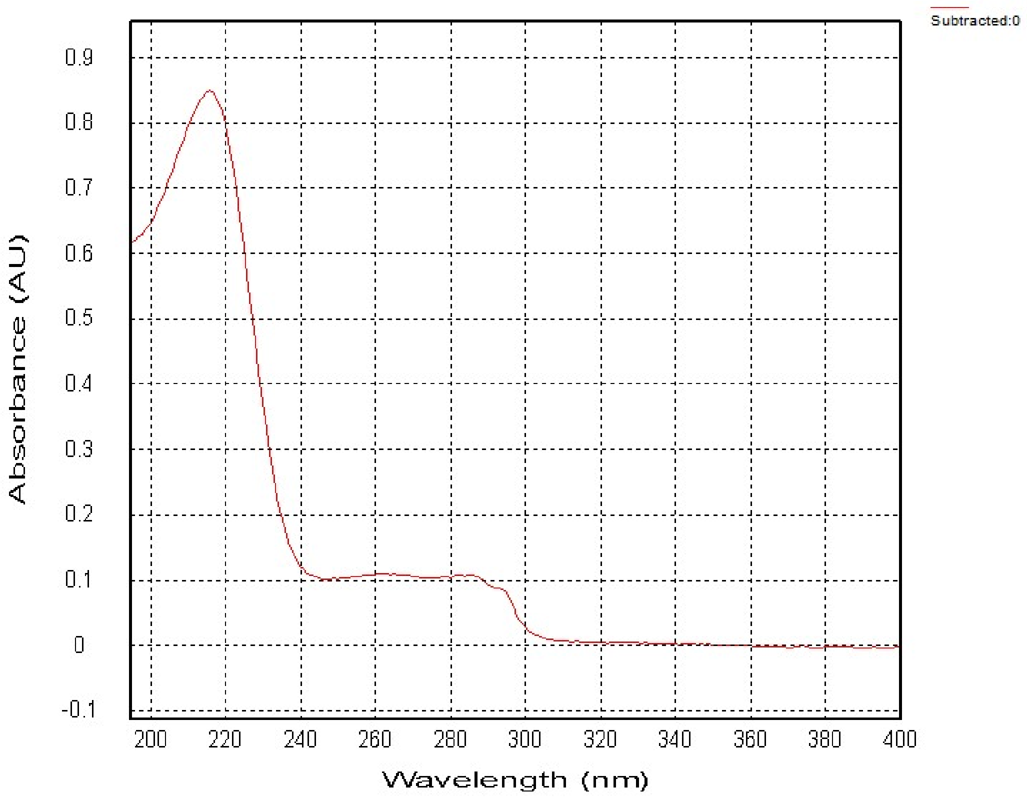

Figure S17. ECD spectrum of compound **2**.

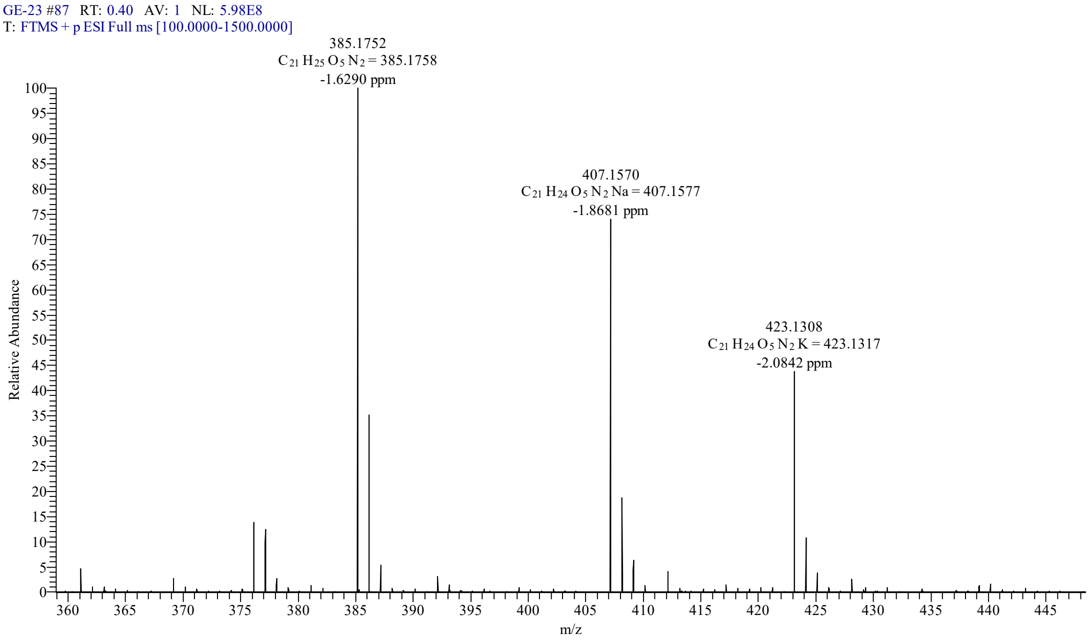

Figure S18. HRESIMS spectrum of compound **2**.

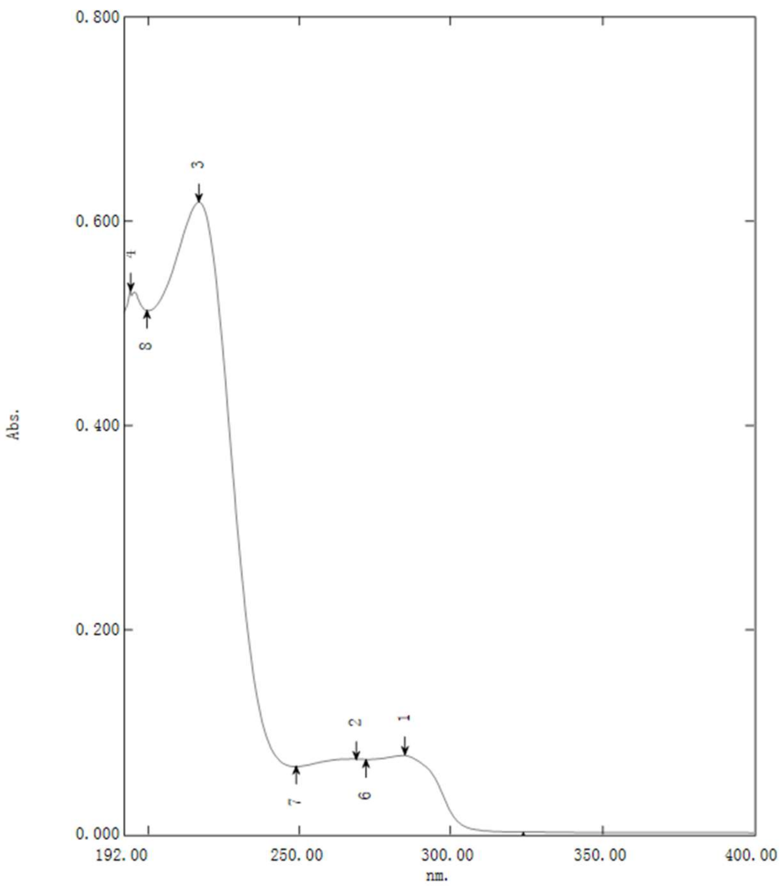

No.	P/V	波长(nm)	Abs.	描述
1	↑	284. 50	0. 077	
2	↑	268. 50	0. 074	
3	↑	216. 50	0. 619	
4	↑	194. 00	0. 532	
5	↓	324. 00	0. 002	
6	↓	272. 00	0. 073	
7	↓	249. 00	0. 066	
8	↓	199. 50	0. 512	

Figure S19. UV spectrum of compound **2**.

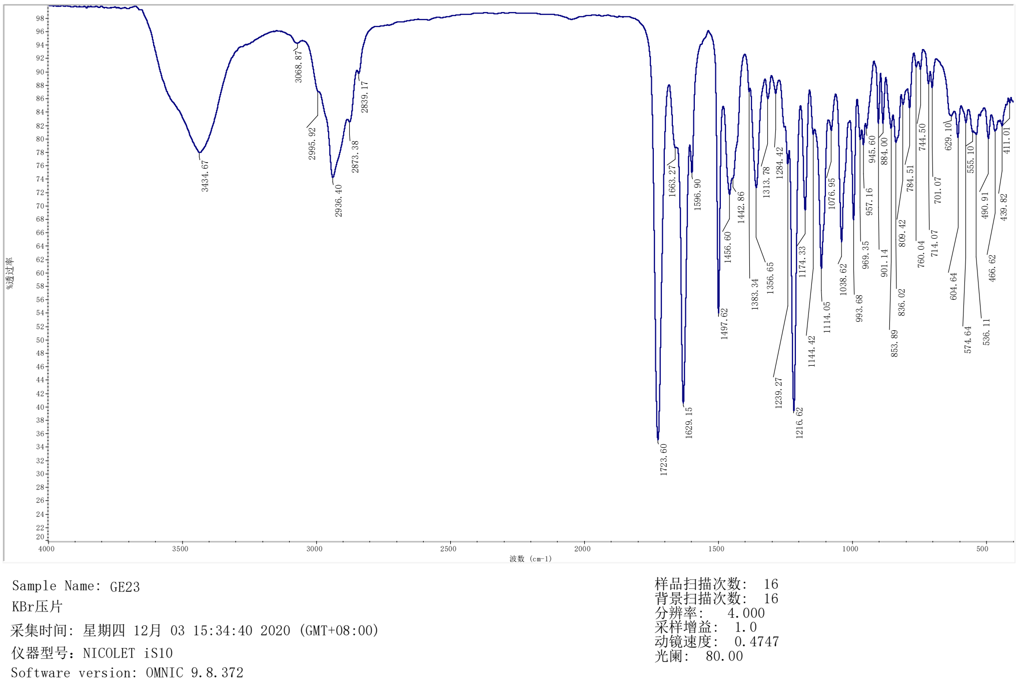

Figure S20. IR spectrum of **2**

3. ECD and ^{13}C NMR calculations of compound 1

3.1. Computational methods

3.1.1. Conformational analysis

Conformational analysis for compound **1A** (**Error! Reference source not found.**) and **1B** (**Error! Reference source not found.**) were performed using systematic algorithm by Confab [19] at MMFF94 force field with RMSD threshold of 0.2 Å and energy window of 7 kcal/mol. The energies of all dominative conformers were provided in **Tables S1** and **S2**.

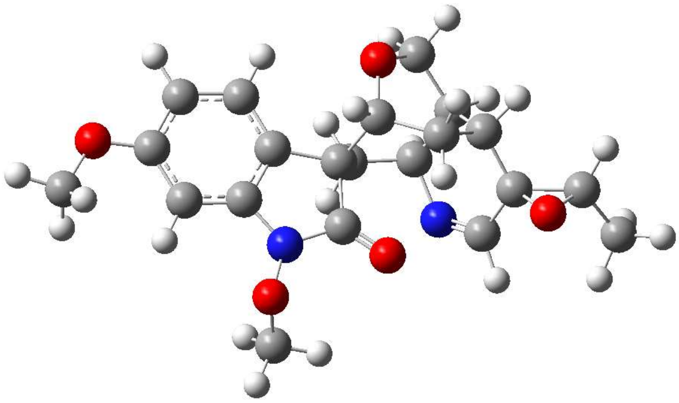

Figure S21. Chemical structure of compounds **1A**.

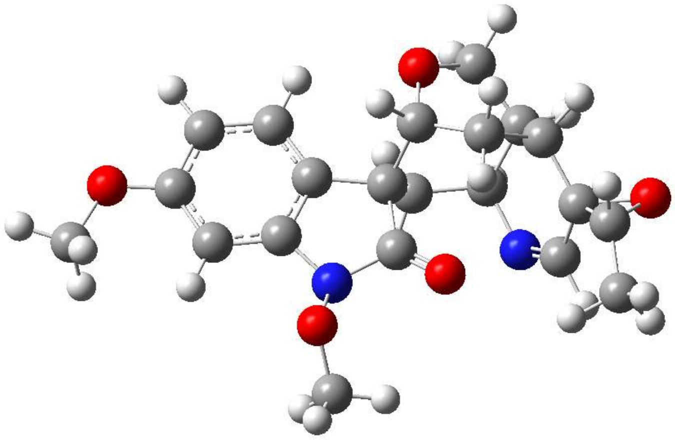

Figure S22. Chemical structure of compounds **1B**.

Table S1 Energies of configurations **1A**.

Copyright: © 2021 by the authors. Licensee MDPI, Basel, Switzerland. This article is an open access article distributed under the terms and conditions of the Creative Commons Attribution (CC BY) license (https://creativecommons.org/licenses/by/4.0/).

Configuration	Conformer	Structure	Energy (kcal/mol)	Population (%)
**1A**	a	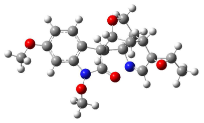	119.23	56.2
**1A**	b	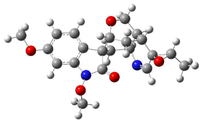	119.74	23.8
**1A**	c	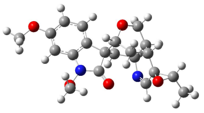	120.10	13.0
**1A**	d	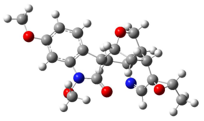	120.46	7.0

Table S2. Energies of configurations **1B**.

Configuration	Conformer	Structure	Energy (kcal/mol)	Population (%)
**1B**	a	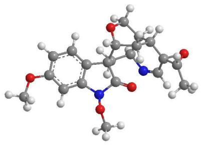	−99.43	43.51
**1B**	b	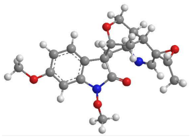	−99.25	32.14
**1B**	c	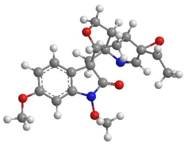	−98.75	13.74
**1B**	d	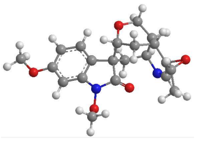	−98.59	10.61

3.1.2. ^{13}C NMR calculations for 1A and 1B

The theoretical calculations were carried out using Gaussian 09 [20]. At first, all conformers were optimized at PM6. Room-temperature equilibrium populations were calculated according to Boltzmann distribution law **Error! Reference source not found.**), based on which dominative conformers of population over 5% were kept. The chosen conformers were further optimized at B3LYP/6-31G(d,p) in gas phase (**Error! Reference source not found.** and **S4**). Vibrational frequency analysis confirmed the stable structures.

$$\frac{N_i}{N} = \frac{g_i e^{-\frac{E_i}{k_B T}}}{\sum g_i e^{-\frac{E_i}{k_B T}}} \quad \textbf{(1)}$$

NMR calculations were carried out following the protocol adapted from Michael *et al.* [21] (**Error! Reference source not found.** and **S6**) using the Gauge-Including Atomic Orbitals (GIAO) method at mPW1PW91/6-311+G(2d,p) level in MeOH simulated by the IEFPCM model. The TMS-corrected NMR chemical shift values were averaged according to Boltzmann distribution and fitted to the experimental values by linear regression. The calculated ^{13}C-NMR and ^{1}H-NMR chemical shift values of TMS in MeOH were 187.3772 ppm and 31.7337 ppm.

3.1.3. ECD calculations for 1A

ECD calculations were conducted at B3LYP/6-311G(d,p) level in methanol with IEFPCM model using Time-dependent Density functional theory (TD-DFT). Rotatory strengths for 30 excited states were calculated. The ECD spectrum was simulated using the ECD/UV analysis tool by overlapping Gaussian functions for each transition according to (2).

$$\Delta\varepsilon(E)=\frac{1}{2.297\times10^{-39}}\times\frac{1}{\sqrt{2\pi\sigma}}\sum_{i}^{A}\Delta E_i R_i e^{-\left(\frac{E-E_i}{2\sigma}\right)^2} \quad \textbf{(2)}$$

where σ represents the width of the band at 1/*e* height, while ΔE_i and R_i are the excitation energies and rotatory strengths for transition *i*, respectively.

The σ and UV-shift values were set 0.27 eV and −13 nm, respectively. The spectrum of the enantiomers were produced directly by mirror inversion about the horizontal axis.

3.2. Energies and Coordinates

3.2.1. Energies at B3LYP theory level

Structures for ^{13}C NMR and ECD calculation were shown in **Error! Reference source not found.** and **S4**.

Table S3. Energies of configurations **1A** at B3LYP/6-311G(d,p) in methanol.

Configuration	Conformer	Structure	E (Hartree)	E (kcal/mol)	Population (%)
**1A**	a	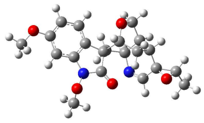	−1300.518383	−816087.6	20.41
**1A**	b	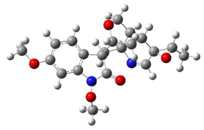	−1300.518481	−816087.6614	22.64
**1A**	c	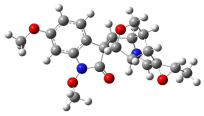	−1300.518633	−816087.7566	26.59
**1A**	d	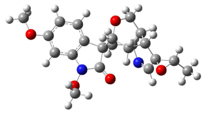	−1300.518758	−816087.835	30.35

Table S4. Energies of configurations **1B** at B3LYP/6-311G(d,p) in methanol.

Configuration	Conformer	Structure	E (Hartree)	E (kcal/mol)	Population (%)
**1B**	a	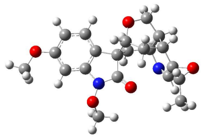	−1300.176358	−815872.98	32.9
**1B**	b	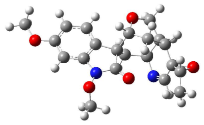	−1300.176541	−815873.09	39.6
**1B**	c	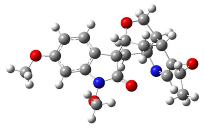	−1300.175408	−815872.38	11.9
**1B**	d	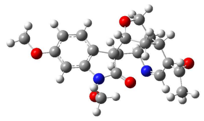	−1300.175659	−815872.54	15.6

3.2.2. Coordinates at B3LYP theory level

Table S5. Standard orientations of configurations **1A** for calculations.

Conformer **1A**-a

Center	Atomic	Atomic	Coordinates (Angstroms)		
Number	Number	Type	X	Y	Z
1	6	0	−3.861956	−1.928437	0.149284
2	6	0	−4.601438	−0.743171	0.010026
3	6	0	−3.954737	0.498596	−0.101449
4	6	0	−2.56634	0.480724	−0.066523
5	6	0	−1.80207	−0.680219	0.067607
6	6	0	−2.466301	−1.898069	0.178544
7	7	0	−1.690683	1.569876	−0.157524

8	6	0	−0.356589	1.199512	−0.234173
9	6	0	−0.314988	−0.331714	0.057509
10	6	0	1.810298	−0.732954	1.623384
11	7	0	2.384096	0.610764	1.726529
12	6	0	3.262012	1.004029	0.894595
13	6	0	3.824592	0.212799	−0.240526
14	6	0	2.97721	−0.975577	−0.654567
15	6	0	2.491714	−1.700637	0.613108
16	6	0	0.277642	−0.625926	1.469147
17	6	0	0.426799	−1.13574	−1.068577
18	6	0	1.778889	−0.590364	−1.564153
19	6	0	1.601509	−2.872402	0.193425
20	8	0	0.521891	−2.511327	−0.678922
21	8	0	0.55471	1.976188	−0.454769
22	6	0	5.288969	0.22024	−0.503118
23	8	0	4.397394	0.996603	−1.30867
24	6	0	6.324301	0.957428	0.310967
25	8	0	−2.135769	2.818649	−0.55197
26	6	0	−1.902508	3.789642	0.4846
27	8	0	−5.957723	−0.895615	−0.011604
28	6	0	−6.763768	0.260439	−0.170751
29	1	0	−4.404299	−2.865033	0.224416
30	1	0	−4.487656	1.431866	−0.226312
31	1	0	−1.902199	−2.821456	0.260997
32	1	0	1.947918	−1.169267	2.623393
33	1	0	3.631408	2.027209	0.995377
34	1	0	3.61644	−1.672979	−1.209497
35	1	0	3.368695	−2.130309	1.116986
36	1	0	−0.151376	−1.579728	1.79071
37	1	0	−0.079962	0.127793	2.178579
38	1	0	−0.254018	−1.155492	−1.924767
39	1	0	1.944791	−1.018494	−2.55795
40	1	0	1.717309	0.491794	−1.690032
41	1	0	2.21808	−3.614634	−0.33424
42	1	0	1.172368	−3.372163	1.073122
43	1	0	5.671648	−0.667717	−1.015697
44	1	0	7.137224	1.29741	−0.341105
45	1	0	6.75708	0.293315	1.068596
46	1	0	5.905737	1.832203	0.813393
47	1	0	−2.323108	4.717731	0.091263
48	1	0	−2.417365	3.504421	1.409119
49	1	0	−0.830534	3.906828	0.661804
50	1	0	−7.79674	−0.092401	−0.167212
51	1	0	−6.620967	0.970176	0.655111
52	1	0	−6.55783	0.769277	−1.121768

Conformer **1A**-b

Center Number	Atomic Number	Atomic Type	Coordinates (Angstroms)		
			X	Y	Z
1	6	0	−3.993783	−1.550049	0.109925
2	6	0	−4.64016	−0.31477	−0.025349

3	6	0	−3.900312	0.87784	−0.133521
4	6	0	−2.523891	0.769506	−0.09733
5	6	0	−1.843155	−0.450249	0.037503
6	6	0	−2.59025	−1.611652	0.141694
7	7	0	−1.568164	1.787725	−0.186899
8	6	0	−0.265029	1.319132	−0.26085
9	6	0	−0.334672	−0.210008	0.036596
10	6	0	1.741567	−0.757277	1.625419
11	7	0	2.413228	0.54046	1.727447
12	6	0	3.326347	0.862164	0.902699
13	6	0	3.840286	0.023894	−0.221627
14	6	0	2.909352	−1.099657	−0.638569
15	6	0	2.358458	−1.779168	0.627386
16	6	0	0.222867	−0.537631	1.455534
17	6	0	0.358612	−1.070125	−1.078615
18	6	0	1.752181	−0.625385	−1.559298
19	6	0	1.386975	−2.883382	0.203571
20	8	0	0.348614	−2.44819	−0.684719
21	8	0	0.700767	2.026035	−0.482993
22	6	0	5.30363	−0.07972	−0.469069
23	8	0	4.480413	0.755031	−1.288841
24	6	0	6.382977	0.585365	0.349902
25	8	0	−1.914416	3.065882	−0.583832
26	6	0	−1.635301	4.014065	0.462169
27	8	0	−5.99529	−0.153925	−0.067802
28	6	0	−6.813143	−1.309737	0.016815
29	1	0	−4.563602	−2.468341	0.184294
30	1	0	−4.406096	1.828037	−0.255634
31	1	0	−2.093929	−2.573493	0.222428
32	1	0	1.836592	−1.196396	2.629163
33	1	0	3.769787	1.855665	1.002219
34	1	0	3.49815	−1.846392	−1.184993
35	1	0	3.195623	−2.270817	1.142304
36	1	0	−0.278491	−1.454857	1.778351
37	1	0	−0.084361	0.244849	2.157367
38	1	0	−0.31224	−1.043034	−1.942464
39	1	0	1.896963	−1.060431	−2.553372
40	1	0	1.770847	0.458894	−1.679573
41	1	0	1.952107	−3.673605	−0.312007
42	1	0	0.911115	−3.343047	1.081227
43	1	0	5.623887	−0.997656	−0.971503
44	1	0	7.226361	0.855628	−0.296065
45	1	0	6.756015	−0.100885	1.119501
46	1	0	6.026042	1.494875	0.837975
47	1	0	−1.977738	4.971275	0.063055
48	1	0	−2.191878	3.765313	1.372839
49	1	0	−0.561961	4.052564	0.665009
50	1	0	−7.842794	−0.951794	−0.040414
51	1	0	−6.622195	−2.000847	−0.814678
52	1	0	−6.665686	−1.840383	0.966738

Conformer **1A**-c

Center Number	Atomic Number	Atomic Type	Coordinates (Angstroms)		
			X	Y	Z
1	6	0	−3.837432	−1.960902	0.016813
2	6	0	−4.598587	−0.781601	0.050675
3	6	0	−3.974136	0.47587	0.081605
4	6	0	−2.585042	0.479168	0.086673
5	6	0	−1.799095	−0.675287	0.076715
6	6	0	−2.442399	−1.90846	0.029787
7	7	0	−1.729803	1.587086	0.083514
8	6	0	−0.387443	1.254023	0.171585
9	6	0	−0.319388	−0.301238	0.120526
10	6	0	1.854692	−1.057705	1.490035
11	7	0	2.475536	0.209124	1.8827
12	6	0	3.353335	0.757218	1.143815
13	6	0	3.864848	0.235309	−0.159617
14	6	0	2.959131	−0.792123	−0.810766
15	6	0	2.48426	−1.782958	0.266172
16	6	0	0.320349	−0.886266	1.415087
17	6	0	0.400187	−0.803832	−1.183597
18	6	0	1.748514	−0.149452	−1.539734
19	6	0	1.55305	−2.806761	−0.385874
20	8	0	0.492363	−2.230996	−1.160143
21	8	0	0.516872	2.066565	0.242949
22	6	0	5.320422	0.260973	−0.46207
23	8	0	4.437717	1.230588	−1.035529
24	6	0	6.40185	0.757499	0.466314
25	8	0	−2.200737	2.865296	0.317693
26	6	0	−1.994687	3.703431	−0.834592
27	8	0	−5.952411	−0.955219	0.04775
28	6	0	−6.77919	0.19622	0.097416
29	1	0	−4.362856	−2.909439	−0.016884
30	1	0	−4.524704	1.406882	0.109974
31	1	0	−1.865007	−2.82652	−0.008234
32	1	0	2.003504	−1.714703	2.359201
33	1	0	3.761865	1.716193	1.471006
34	1	0	3.546098	−1.355307	−1.546544
35	1	0	3.361006	−2.335132	0.632133
36	1	0	−0.12241	−1.877349	1.554297
37	1	0	0.010285	−0.287526	2.27769
38	1	0	−0.294153	−0.603593	−2.005564
39	1	0	1.887981	−0.267008	−2.619039
40	1	0	1.702059	0.923001	−1.340862
41	1	0	2.140315	−3.441928	−1.064366
42	1	0	1.106793	−3.465817	0.372457
43	1	0	5.658064	−0.492802	−1.180044
44	1	0	7.206084	1.222892	−0.115284
45	1	0	6.834746	−0.078472	1.028479
46	1	0	6.026283	1.496922	1.177012
47	1	0	−2.437875	4.662826	−0.558879
48	1	0	−0.926497	3.823933	−1.030529
49	1	0	−2.505205	3.29147	−1.712676

50	1	0	−7.806468	−0.172818	0.095986
51	1	0	−6.604457	0.779902	1.010863
52	1	0	−6.625473	0.842286	−0.77726

Conformer **1A**-d

Center	Atomic	Atomic	Coordinates (Angstroms)		
Number	Number	Type	X	Y	Z
1	6	0	−3.973065	−1.583492	0.024772
2	6	0	−4.640647	−0.35259	0.062163
3	6	0	−3.921126	0.856327	0.102325
4	6	0	−2.542634	0.767824	0.10859
5	6	0	−1.840624	−0.447333	0.091451
6	6	0	−2.568712	−1.624116	0.039828
7	7	0	−1.604881	1.8051	0.113095
8	6	0	−0.290488	1.3725	0.182467
9	6	0	−0.337199	−0.183715	0.130248
10	6	0	1.783758	−1.09284	1.490058
11	7	0	2.493735	0.127175	1.879231
12	6	0	3.404347	0.611978	1.135841
13	6	0	3.871464	0.055036	−0.169761
14	6	0	2.892968	−0.906264	−0.816483
15	6	0	2.355501	−1.860972	0.26316
16	6	0	0.264502	−0.814675	1.421673
17	6	0	0.337679	−0.738493	−1.176393
18	6	0	1.726915	−0.181831	−1.540818
19	6	0	1.353079	−2.817687	−0.384978
20	8	0	0.327731	−2.169155	−1.149544
21	8	0	0.672698	2.115036	0.242982
22	6	0	5.324056	−0.02345	−0.477765
23	8	0	4.510473	1.006338	−1.04861
24	6	0	6.441281	0.395601	0.446443
25	8	0	−1.974012	3.116455	0.339791
26	6	0	−1.736787	3.923508	−0.828679
27	8	0	−5.998549	−0.211342	0.05935
28	6	0	−6.797258	−1.38294	0.019163
29	1	0	−4.527369	−2.513288	−0.015442
30	1	0	−4.443341	1.804955	0.13367
31	1	0	−2.059343	−2.581518	−0.002532
32	1	0	1.890056	−1.758713	2.35875
33	1	0	3.881243	1.539729	1.460372
34	1	0	3.436368	−1.509297	−1.554122
35	1	0	3.193303	−2.472949	0.62528
36	1	0	−0.245433	−1.772703	1.561331
37	1	0	0.00048	−0.197	2.286286
38	1	0	−0.344987	−0.491626	−1.995384
39	1	0	1.852447	−0.310226	−2.620604
40	1	0	1.756663	0.891313	−1.342974
41	1	0	1.892412	−3.488009	−1.069408
42	1	0	0.868366	−3.448071	0.374153
43	1	0	5.604326	−0.799887	−1.196345
44	1	0	7.276575	0.797786	−0.138509

45	1	0	6.812405	−0.467407	1.011939
46	1	0	6.122684	1.164227	1.153944
47	1	0	−2.105231	4.914861	−0.556392
48	1	0	−0.667553	3.966401	−1.050558
49	1	0	−2.296141	3.53791	−1.688635
50	1	0	−7.833053	−1.038538	0.027137
51	1	0	−6.615271	−1.964752	−0.894062
52	1	0	−6.620371	−2.022025	0.894231

Table S6. Standard orientations of configurations **1B** for calculations.

Conformer **1B**-a

Center	Atomic	Atomic	Coordinates (Angstroms)		
Number	Number	Type	X	Y	Z
1	6	0	−3.907945	−1.79092	−0.057005
2	6	0	−4.534445	−0.535281	−0.111326
3	6	0	−3.776126	0.647346	−0.10973
4	6	0	−2.396665	0.499046	−0.050987
5	6	0	−1.744355	−0.733533	0.002127
6	6	0	−2.516578	−1.891109	0.000863
7	7	0	−1.421527	1.506546	−0.036469
8	6	0	−0.127775	1.019173	−0.117769
9	6	0	−0.233293	−0.524218	0.054789
10	6	0	0.275415	−0.988829	1.452708
11	6	0	1.791883	−1.171646	1.680345
12	7	0	2.401365	0.101933	2.068027
13	6	0	3.221472	0.68116	1.287222
14	6	0	3.667336	0.21401	−0.055941
15	6	0	3.004799	−1.050781	−0.5913
16	6	0	2.536399	−1.953753	0.568476
17	6	0	1.70961	−3.110614	0.002053
18	8	0	0.547167	−2.700472	−0.735805
19	6	0	0.477244	−1.318446	−1.100857
20	6	0	1.837826	−0.800543	−1.587168
21	6	0	4.310542	1.147056	−1.007531
22	6	0	4.45931	2.637733	−0.823646
23	8	0	5.119685	0.275105	−0.213556
24	8	0	0.857707	1.721991	−0.267555
25	1	0	4.249794	0.8377	−2.055715
26	8	0	−1.737906	2.818636	−0.338021
27	6	0	−1.468683	3.675101	0.787165
28	8	0	−5.897558	−0.560938	−0.168419
29	6	0	−6.593509	0.673498	−0.228373
30	1	0	−4.534218	−2.676709	−0.067405
31	1	0	−4.219697	1.632467	−0.168148

32	1	0	−2.039502	−2.866138	0.022902
33	1	0	−0.200793	−1.956581	1.633424
34	1	0	−0.101921	−0.306932	2.222361
35	1	0	1.851174	−1.788732	2.588333
36	1	0	3.663012	1.614183	1.641835
37	1	0	3.796189	−1.588036	−1.126449
38	1	0	3.429557	−2.390939	1.034704
39	1	0	2.34626	−3.714891	−0.661959
40	1	0	1.357102	−3.769191	0.806348
41	1	0	−0.206354	−1.299727	−1.955436
42	1	0	2.045838	−1.342831	−2.516818
43	1	0	1.756635	0.257081	−1.842648
44	1	0	5.321885	3.000079	−1.394705
45	1	0	3.562865	3.150908	−1.188917
46	1	0	4.613556	2.90758	0.223348
47	1	0	−1.794017	4.665938	0.462602
48	1	0	−2.043099	3.358115	1.664828
49	1	0	−0.399028	3.684226	1.011684
50	1	0	−7.653952	0.417426	−0.264538
51	1	0	−6.399825	1.289954	0.659593
52	1	0	−6.327839	1.244421	−1.127968

Conformer **1B**-b

Center Number	Atomic Number	Atomic Type	Coordinates (Angstroms)		
			X	Y	Z
1	6	0	−4.039732	−1.353265	−0.043562
2	6	0	−4.553162	−0.052131	−0.120671
3	6	0	−3.693263	1.062699	−0.136422
4	6	0	−2.337287	0.810563	−0.069452
5	6	0	−1.789088	−0.478169	0.008531
6	6	0	−2.651491	−1.561184	0.021602
7	7	0	−1.277917	1.72703	−0.072006
8	6	0	−0.029555	1.131439	−0.125202
9	6	0	−0.265915	−0.395524	0.06215
10	6	0	0.203531	−0.887255	1.464409
11	6	0	1.698321	−1.20292	1.692254
12	7	0	2.42259	0.013893	2.062723
13	6	0	3.29678	0.501854	1.278225
14	6	0	3.700078	−0.025248	−0.056416
15	6	0	2.915424	−1.223187	−0.578638
16	6	0	2.36436	−2.065601	0.589516
17	6	0	1.432509	−3.145325	0.034525
18	8	0	0.318465	−2.637001	−0.716392

19	6	0	0.374117	−1.255909	−1.087696
20	6	0	1.776071	−0.863115	−1.573027
21	6	0	4.433704	0.827834	−1.017748
22	6	0	4.726527	2.299115	−0.851543
23	8	0	5.151313	−0.1092	−0.209562
24	8	0	1.015395	1.745081	−0.263702
25	1	0	4.345818	0.512413	−2.062158
26	8	0	−1.475147	3.060142	−0.376632
27	6	0	−1.171084	3.890343	0.759549
28	8	0	−5.882227	0.250443	−0.189482
29	6	0	−6.817719	−0.816064	−0.195117
30	1	0	−4.701791	−2.21052	−0.038432
31	1	0	−4.096397	2.065268	−0.213312
32	1	0	−2.257162	−2.572023	0.060713
33	1	0	−0.356467	−1.806263	1.65906
34	1	0	−0.108502	−0.165207	2.226708
35	1	0	1.703241	−1.81062	2.608572
36	1	0	3.823122	1.394126	1.621552
37	1	0	3.647331	−1.84048	−1.112093
38	1	0	3.212588	−2.580123	1.060721
39	1	0	2.010939	−3.816932	−0.617766
40	1	0	1.015663	−3.755439	0.846386
41	1	0	−0.303296	−1.180118	−1.944049
42	1	0	1.93424	−1.417322	−2.505396
43	1	0	1.791266	0.198762	−1.823058
44	1	0	5.616409	2.571108	−1.430793
45	1	0	3.881219	2.892309	−1.217655
46	1	0	4.912398	2.563516	0.191685
47	1	0	−1.399577	4.905176	0.426946
48	1	0	−1.800253	3.624654	1.616352
49	1	0	−0.112484	3.807887	1.019377
50	1	0	−7.802054	−0.348998	−0.261592
51	1	0	−6.670643	−1.477466	−1.059045
52	1	0	−6.759472	−1.40959	0.726822

Conformer **1B**-c

Center Number	Atomic Number	Atomic Type	Coordinates (Angstroms)		
			X	Y	Z
1	6	0	−3.908267	−1.782756	−0.119248
2	6	0	−4.540598	−0.534128	−0.004852
3	6	0	−3.786722	0.645905	0.108634
4	6	0	−2.40548	0.501027	0.106766
5	6	0	−1.747192	−0.726289	0.014918

6	6	0	−2.515529	−1.879856	−0.107065
7	7	0	−1.433878	1.507695	0.176162
8	6	0	−0.135423	1.032949	0.182437
9	6	0	−0.236858	−0.515848	0.078986
10	6	0	0.305667	−1.224464	1.355845
11	6	0	1.824682	−1.467037	1.507242
12	7	0	2.465888	−0.302591	2.119516
13	6	0	3.280214	0.406605	1.448146
14	6	0	3.689954	0.202087	0.029263
15	6	0	2.991762	−0.925549	−0.723216
16	6	0	2.535637	−2.029806	0.250741
17	6	0	1.685427	−3.050528	−0.50904
18	8	0	0.517535	−2.497315	−1.136546
19	6	0	0.45109	−1.07201	−1.221631
20	6	0	1.805555	−0.466921	−1.617194
21	6	0	4.330175	1.291851	−0.740868
22	6	0	4.514455	2.715938	−0.275845
23	8	0	5.139034	0.271029	−0.1504
24	8	0	0.855751	1.743696	0.22807
25	1	0	4.241681	1.191813	−1.827425
26	8	0	−1.760438	2.826085	0.420952
27	6	0	−1.528118	3.63716	−0.746191
28	8	0	−5.904693	−0.563387	−0.016763
29	6	0	−6.60722	0.6631	0.101612
30	1	0	−4.531353	−2.665757	−0.213891
31	1	0	−4.235603	1.625837	0.203219
32	1	0	−2.035848	−2.848848	−0.204765
33	1	0	−0.181933	−2.203298	1.378041
34	1	0	−0.038342	−0.686307	2.245532
35	1	0	1.890531	−2.248458	2.277766
36	1	0	3.745354	1.246274	1.967361
37	1	0	3.758732	−1.358984	−1.375341
38	1	0	3.432728	−2.555078	0.60514
39	1	0	2.303731	−3.524232	−1.286505
40	1	0	1.336728	−3.845808	0.162469
41	1	0	−0.246165	−0.883501	−2.044538
42	1	0	1.99099	−0.796345	−2.646019
43	1	0	1.727942	0.62141	−1.637231
44	1	0	5.373816	3.166913	−0.785388
45	1	0	3.623082	3.30723	−0.513283
46	1	0	4.693553	2.775091	0.799778
47	1	0	−1.863261	4.635879	−0.458242

48	1	0	−0.463364	3.652984	−0.991771
49	1	0	−2.116048	3.273678	−1.59677
50	1	0	−7.66724	0.40453	0.071694
51	1	0	−6.38373	1.16721	1.051132
52	1	0	−6.376845	1.3426	−0.729856

Conformer **1B**-d

Center Number	Atomic Number	Atomic Type	Coordinates (Angstroms)		
			X	Y	Z
1	6	0	−4.037037	−1.345547	−0.089794
2	6	0	−4.553827	−0.049503	0.035832
3	6	0	−3.695755	1.060481	0.154179
4	6	0	−2.338115	0.807744	0.143889
5	6	0	−1.786321	−0.477529	0.038919
6	6	0	−2.647441	−1.554539	−0.084944
7	7	0	−1.280253	1.721591	0.211744
8	6	0	−0.027996	1.136686	0.190333
9	6	0	−0.262604	−0.397998	0.090921
10	6	0	0.225273	−1.147447	1.366558
11	6	0	1.718148	−1.521282	1.507587
12	7	0	2.463423	−0.416417	2.112592
13	6	0	3.331321	0.217592	1.43301
14	6	0	3.708117	−0.022506	0.010527
15	6	0	2.911517	−1.08926	−0.73325
16	6	0	2.368319	−2.146557	0.248466
17	6	0	1.426806	−3.092494	−0.500016
18	8	0	0.306397	−2.442508	−1.121883
19	6	0	0.363366	−1.016333	−1.211565
20	6	0	1.762614	−0.535199	−1.62199
21	6	0	4.427542	1.010594	−0.767577
22	6	0	4.730076	2.415506	−0.30614
23	8	0	5.156037	−0.072936	−0.184315
24	8	0	1.021051	1.759794	0.216654
25	1	0	4.31953	0.917591	−1.852957
26	8	0	−1.482651	3.063952	0.45537
27	6	0	−1.231729	3.844525	−0.728577
28	8	0	−5.884628	0.252646	0.050149
29	6	0	−6.818492	−0.808535	−0.071332
30	1	0	−4.69805	−2.197786	−0.190081
31	1	0	−4.101929	2.059952	0.253015
32	1	0	−2.252435	−2.560139	−0.192076
33	1	0	−0.345521	−2.079853	1.395171
34	1	0	−0.064231	−0.578959	2.256756

35	1	0	1.72104	−2.303985	2.279665
36	1	0	3.872981	1.013865	1.946292
37	1	0	3.634854	−1.58906	−1.387715
38	1	0	3.21905	−2.746542	0.598581
39	1	0	1.995088	−3.622041	−1.279781
40	1	0	1.016011	−3.851888	0.177977
41	1	0	−0.321422	−0.770944	−2.029881
42	1	0	1.909403	−0.888618	−2.649117
43	1	0	1.780527	0.555567	−1.651848
44	1	0	5.6097	2.801248	−0.834222
45	1	0	3.880604	3.072657	−0.523162
46	1	0	4.936947	2.458182	0.765284
47	1	0	−1.470012	4.86964	−0.437288
48	1	0	−0.180321	3.769442	−1.017715
49	1	0	−1.88351	3.526692	−1.550231
50	1	0	−7.805161	−0.343168	−0.034588
51	1	0	−6.703146	−1.340246	−1.025007
52	1	0	−6.724541	−1.5258	0.754565

3.3. Experimental and computed NMR chemical shifts

3.3.1. ^{13}C-NMR chemical shifts

The TMS-corrected computed ^{13}C-NMR chemical shifts of compounds **1A** and **1B** were fitted to the experimental values by Ordinary Least Squares (OLS) Linear Regression method in order to remove systematic error that results from the conformational search and random error from experimental conditions (**Error! Reference source not found.** and **S8**).

Table S7. Experimental and computed chemical shifts of **1A**.

^{13}C-NMR:

**Position**	**Experimental**	**Calculated-1A**	**Fitted-1A**
2	174.39	180.1308884	174.86
3	74.84	78.27315228	75.37
5	60.72	65.29904352	62.70
6	37.07	41.22994124	39.19
7	57.43	61.44131996	58.93
8	125.07	129.648829	125.55
9	126.97	131.1750764	127.04
10	109.3	110.2297935	106.58
11	162.09	166.3845379	161.43
12	95.42	96.12810684	92.81
13	140.59	145.8881386	141.41

14	25.02	26.15250744	24.47
15	31.28	34.675576	32.79
16	34.23	37.6839056	35.73
17	67.89	69.78754964	67.09
18	14.67	15.8765326	14.43
19	64.61	68.60823892	65.93
20	63.38	65.28327652	62.69
21	165.17	171.9846543	166.90
NOMe	64.15	63.86602044	61.30
ArOMe	56.21	55.65657172	53.28

Table S8. Experimental and computed chemical shifts of **1B**.

^{13}C-NMR:

Position	Experimental	Calculated-1B	Fitted-1B
2	174.39	180.3678	175.63
3	74.84	79.75143	77.26
5	60.72	64.60605	62.45
6	37.07	39.82705	38.22
7	57.43	61.71209	59.62
8	125.07	129.9134	126.30
9	126.97	130.4208	126.80
10	109.3	110.2777	107.10
11	162.09	166.3813	161.95
12	95.42	96.329	93.46
13	140.59	145.5407	141.58
14	25.02	30.33824	28.94
15	31.28	36.897	35.36
16	34.23	35.6741	34.16
17	67.89	68.73975	66.49
18	14.67	14.72597	13.68
19	64.61	63.37095	61.24
20	63.38	61.43025	59.34
21	165.17	170.0837	165.57
NOMe	64.15	63.77054	61.63

ArOMe	56.21	55.66953	53.71

Relatively higher R^2 and lower CMAD and CLAD values were shown in both ^{13}C-NMR Ordinary Least Squares Linear Regression (OLS-LR) for compound **1**, which indicated that this configuration was the correct structure (**Table S9**).

Table S9. Statistics of Ordinary Least Squares Linear Regression (OLS-LR) of experimental and computed ^{13}C-NMR chemical shifts.

Type	Compound	CMAD[a]	CLAD[b]	R^2	*RMSD*	*F*	*p* value
^{13}C NMR	**1A**	1.34	2.93	0.9989	1.6885	16697.69	< 0.01
	**1B**	1.84	4.08	0.9978	2.3288	8768.73	< 0.01

[a] CMAD = corrected mean absolute deviation, computed as $(1/n)\sum_i^n |\delta_{calc} - \delta_{exp}|$, where δ_{calc} and δ_{exp} refer to the calculated and experimental chemical shifts. [b] CLAD = corrected largest absolute deviation, computed as $\max(|\delta_{calc} - \delta_{exp}|)$.

4. ECD and ^{13}C NMR calculations of compound 2

4.1. Computational methods

4.1.1. Conformational analysis

Conformational analysis for compound **2A** (**Error! Reference source not found.**) and **2B** (**Error! Reference source not found.**) were performed using systematic algorithm by Confab [19] at MMFF94 force field with RMSD threshold of 0.2 Å and energy window of 7 kcal/mol. The energies of all dominative conformers were provided in **Tables S10** and **S11**.

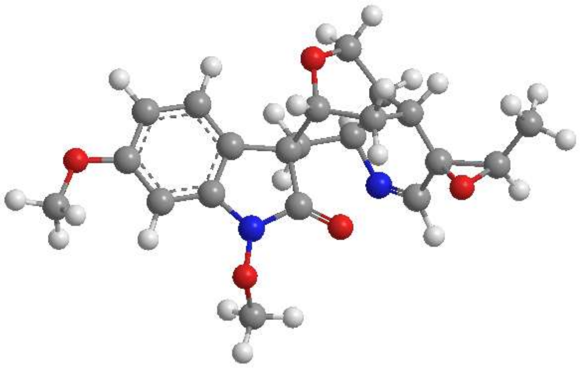

Figure S23. Chemical structure of compounds **2A**.

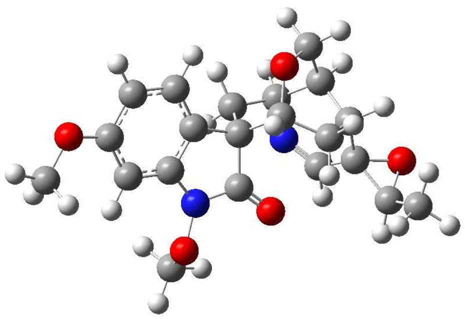

Figure S24. Chemical structure of compounds **2B**.

Table S10. Energies of configurations **2A**.

Configuration	Conformer	Structure	Energy (kcal/mol)	Population (%)
**2A**	a	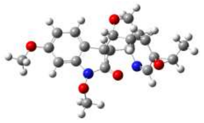	120.35	47.3
**2A**	b	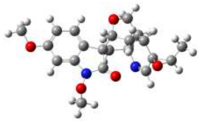	120.75	24.1
**2A**	c	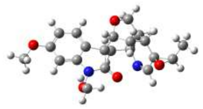	120.9	18.7
**2A**	d	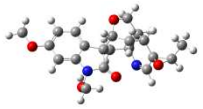	121.27	10

Table S11. Energies of configurations **2B**.

Configuration	Conformer	Structure	Energy (kcal/mol)	Population (%)
**2B**	a	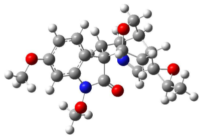	116.64	55.7
**2B**	b	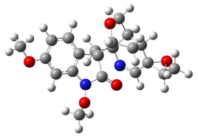	116.92	34.7
**2B**	c	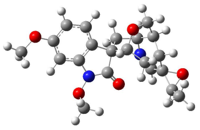	117.99	5.7
**2B**	d	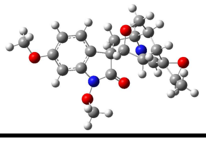	118.22	3.9

4.1.2. ^{13}C NMR calculations for 2A and 2B

The theoretical calculations were carried out using Gaussian 09 [20]. At first, all conformers were optimized at PM6. Room-temperature equilibrium populations were calculated according to Boltzmann distribution law **Error! Reference source not found.**), based on which dominative conformers of population over 5% were kept. The chosen conformers were further optimized at B3LYP/6-31G(d,p) in gas phase (**Error! Reference source not found.** and **S13**). Vibrational frequency analysis confirmed the stable structures.

NMR calculations were carried out following the protocol adapted from Michael *et al*. [21] (**Error! Reference source not found.** and **S15**) using the Gauge-Including Atomic Orbitals (GIAO) method at mPW1PW91/6-311+G(2d,p) level in MeOH simulated by the

IEFPCM model. The TMS-corrected NMR chemical shift values were averaged according to Boltzmann distribution and fitted to the experimental values by linear regression. The calculated ^{13}C-NMR and ^{1}H-NMR chemical shift values of TMS in MeOH were 187.3772 ppm and 31.7337 ppm.

4.1.3. ECD calculations for 2A

ECD calculations were conducted at B3LYP/6-311G(d,p) level in methanol with IEFPCM model using Time-dependent Density functional theory (TD-DFT). Rotatory strengths for 30 excited states were calculated. The ECD spectrum was simulated using the ECD/UV analysis tool by overlapping Gaussian functions for each transition according to (2).

4.2. Energies and Coordinates

4.2.1. Energies at B3LYP theory level

Structures for ^{13}C NMR and ECD calculation were shown in **Error! Reference source not found.** and **S13**.

Table S12. Energies of configurations **2A** at B3LYP/6-311G(d,p) in methanol.

Configuration	Conformer	Structure	E (Hartree)	E (kcal/mol)	Population (%)
**2A**	a	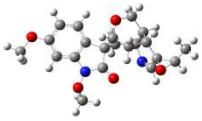	−1300.175437	−815872.4	27.45
**2A**	b	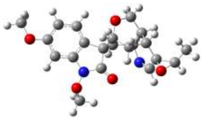	−1300.17555	−815872.47	30.95
**2A**	c	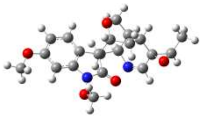	−1300.175149	−815872.22	20.24
**2A**	d	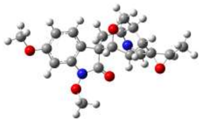	−1300.1752	−815872.25	21.36

Table S13. Energies of configurations **2B** at B3LYP/6−311G(d,p) in methanol.

Configuration	Conformer	Structure	E (Hartree)	E (kcal/mol)	Population (%)
**2B**	a	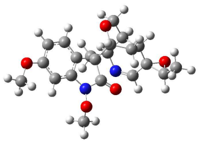	−1300.52	−816087	21.04
**2B**	b	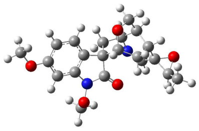	−1300.52	−816087	26.02
**2B**	c	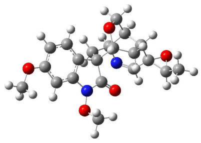	−1300.52	−816087	24.26
**2B**	d	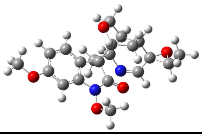	−1300.52	−816087	28.67

4.2.2. Coordinates at B3LYP theory level

Table S14. Standard orientations of configurations **2A** for calculations.

Conformer **2A**-a

Center Number	Atomic Number	Atomic Type	Coordinates (Angstroms) X	Y	Z
1	6	0	−3.774922	−2.033273	0.060978
2	6	0	−4.55433	−0.877221	−0.104114
3	6	0	−3.953001	0.389413	−0.18711
4	6	0	−2.567434	0.42575	−0.0981
5	6	0	−1.764044	−0.7051	0.062125
6	6	0	−2.383683	−1.948428	0.14439
7	7	0	−1.732573	1.54884	−0.150529
8	6	0	−0.382956	1.231878	−0.172377
9	6	0	−0.292695	−0.298319	0.111732
10	6	0	0.255165	−0.579282	1.543703
11	6	0	1.783242	−0.6151	1.762944
12	7	0	2.292161	0.749555	1.91903
13	6	0	3.162386	1.201776	1.110991
14	6	0	3.788261	0.480338	−0.039237
15	6	0	3.045098	−0.765074	−0.481799
16	6	0	2.555141	−1.524598	0.76638
17	6	0	1.749587	−2.74202	0.306689
18	8	0	0.674108	−2.434941	−0.591163
19	6	0	0.524343	−1.066184	−0.987991
20	6	0	1.862625	−0.456203	−1.441252
21	6	0	5.247028	0.694924	−0.246314
22	8	0	4.286988	1.362793	−1.06879

23	8	0	0.506011	2.04502	−0.348139
24	6	0	6.182511	−0.282337	−0.914163
25	8	0	−2.210796	2.781454	−0.556779
26	6	0	−2.054859	3.755901	0.491183
27	8	0	−5.901752	−1.082247	−0.178729
28	6	0	−6.745071	0.042258	−0.367928
29	1	0	−4.282665	−2.990589	0.113168
30	1	0	−4.516747	1.301841	−0.329459
31	1	0	−1.787446	−2.849343	0.247572
32	1	0	−0.139893	−1.556832	1.836361
33	1	0	−0.168648	0.147521	2.244806
34	1	0	1.89855	−1.068178	2.758295
35	1	0	3.502338	2.232988	1.248487
36	1	0	3.739855	−1.428679	−1.006132
37	1	0	3.437433	−1.908064	1.29755
38	1	0	2.424434	−3.440103	−0.210254
39	1	0	1.325698	−3.276826	1.167963
40	1	0	−0.126129	−1.120071	−1.866044
41	1	0	2.087267	−0.887873	−2.422099
42	1	0	1.749465	0.619295	−1.584668
43	1	0	5.73898	1.360615	0.469343
44	1	0	6.971624	0.266005	−1.441639
45	1	0	6.663475	−0.928173	−0.169694
46	1	0	5.666317	−0.90816	−1.646266
47	1	0	−2.497928	4.668214	0.085803
48	1	0	−2.591254	3.445596	1.395123
49	1	0	−0.995662	3.915001	0.70787
50	1	0	−7.762942	−0.350219	−0.405789
51	1	0	−6.66265	0.754597	0.463918
52	1	0	−6.521427	0.561443	−1.309298

Conformer **2A**-b

Center Number	Atomic Number	Atomic Type	Coordinates (Angstroms)		
			X	Y	Z
1	6	0	−3.913905	−1.66202	0.042844
2	6	0	−4.604771	−0.456663	−0.135471
3	6	0	−3.911378	0.764916	−0.227027
4	6	0	−2.534364	0.714918	−0.132088
5	6	0	−1.809819	−0.473683	0.046126
6	6	0	−2.511559	−1.664267	0.133968
7	7	0	−1.618518	1.770886	−0.196438
8	6	0	−0.295163	1.357104	−0.208541
9	6	0	−0.313597	−0.170761	0.099253
10	6	0	0.204985	−0.465787	1.539648
11	6	0	1.724332	−0.615	1.770165
12	7	0	2.336177	0.708935	1.908199
13	6	0	3.244738	1.079208	1.100501
14	6	0	3.822114	0.291386	−0.031619
15	6	0	2.985405	−0.897492	−0.461477
16	6	0	2.430258	−1.597294	0.793965
17	6	0	1.537345	−2.756624	0.345751
18	8	0	0.496364	−2.382846	−0.567123

19	6	0	0.452364	−1.011861	−0.98263
20	6	0	1.835732	−0.508393	−1.431892
21	6	0	5.29537	0.386061	−0.225154
22	8	0	4.39983	1.113145	−1.069874
23	8	0	0.650076	2.101566	−0.393272
24	6	0	6.157734	−0.673925	−0.864541
25	8	0	−2.000666	3.03146	−0.61511
26	6	0	−1.805507	3.99513	0.436076
27	8	0	−5.962316	−0.353527	−0.237838
28	6	0	−6.733852	−1.542426	−0.181255
29	1	0	−4.448037	−2.602352	0.105713
30	1	0	−4.450504	1.691703	−0.381853
31	1	0	−1.979409	−2.603368	0.247889
32	1	0	−0.26562	−1.404282	1.847633
33	1	0	−0.165218	0.304202	2.224821
34	1	0	1.798465	−1.058883	2.77353
35	1	0	3.662217	2.083326	1.224446
36	1	0	3.627744	−1.621726	−0.972223
37	1	0	3.276922	−2.039014	1.33775
38	1	0	2.161042	−3.512422	−0.153989
39	1	0	1.066714	−3.243558	1.211292
40	1	0	−0.193436	−1.029028	−1.865644
41	1	0	2.034073	−0.962844	−2.408075
42	1	0	1.803588	0.571528	−1.58331
43	1	0	5.830868	1.02338	0.485063
44	1	0	6.992715	−0.199346	−1.392973
45	1	0	6.579071	−1.340797	−0.102697
46	1	0	5.601399	−1.271564	−1.590703
47	1	0	−2.168639	4.935821	0.016543
48	1	0	−2.39053	3.728141	1.323449
49	1	0	−0.744363	4.077956	0.684568
50	1	0	−7.77374	−1.229072	−0.290881
51	1	0	−6.474054	−2.230865	−0.996146
52	1	0	−6.609961	−2.059241	0.779598
Conformer **2A**-c					
Center Number	Atomic Number	Atomic Type	Coordinates (Angstroms)		
			X	Y	Z
1	6	0	−3.773098	−2.02478	−0.101546
2	6	0	−4.56043	−0.862348	−0.087708
3	6	0	−3.965375	0.407747	−0.016297
4	6	0	−2.57821	0.440554	0.049153
5	6	0	−1.767916	−0.696905	0.061282
6	6	0	−2.381336	−1.942895	−0.0269
7	7	0	−1.747674	1.566158	0.094973
8	6	0	−0.403238	1.260409	0.234998
9	6	0	−0.299968	−0.292171	0.174269
10	6	0	0.290219	−0.875853	1.492235
11	6	0	1.82289	−0.996999	1.64872
12	7	0	2.381229	0.276167	2.109152
13	6	0	3.248371	0.88501	1.408092
14	6	0	3.822388	0.444655	0.098804

15	6	0	3.026418	−0.64009	−0.600504
16	6	0	2.54793	−1.662782	0.447352
17	6	0	1.704068	−2.724535	−0.26096
18	8	0	0.634566	−2.196006	−1.056971
19	6	0	0.490355	−0.773222	−1.09654
20	6	0	1.825357	−0.070456	−1.404286
21	6	0	5.278778	0.673245	−0.105503
22	8	0	4.31308	1.537273	−0.710903
23	8	0	0.480105	2.090741	0.350408
24	6	0	6.165249	−0.135083	−1.020274
25	8	0	−2.255364	2.831804	0.319566
26	6	0	−2.017284	3.685483	−0.815122
27	8	0	−5.908795	−1.064557	−0.150923
28	6	0	−6.761651	0.068425	−0.122656
29	1	0	−4.275873	−2.983791	−0.168201
30	1	0	−4.536478	1.326615	−0.001646
31	1	0	−1.782452	−2.847696	−0.049021
32	1	0	−0.125163	−1.883423	1.592079
33	1	0	−0.086951	−0.302617	2.345495
34	1	0	1.946027	−1.674969	2.505642
35	1	0	3.626556	1.842447	1.779848
36	1	0	3.678994	−1.175759	−1.297055
37	1	0	3.433031	−2.175173	0.849494
38	1	0	2.353025	−3.307872	−0.93031
39	1	0	1.271559	−3.426631	0.465663
40	1	0	−0.17659	−0.603811	−1.94759
41	1	0	2.021081	−0.209965	−2.47243
42	1	0	1.72597	1.003493	−1.237272
43	1	0	5.811006	1.139717	0.72898
44	1	0	6.947434	0.509194	−1.438273
45	1	0	6.657276	−0.943546	−0.466358
46	1	0	5.607365	−0.565652	−1.855274
47	1	0	−2.494061	4.631992	−0.551197
48	1	0	−0.944448	3.831639	−0.961957
49	1	0	−2.478752	3.270596	−1.718604
50	1	0	−7.779736	−0.322211	−0.171599
51	1	0	−6.637885	0.644064	0.804106
52	1	0	−6.585641	0.728594	−0.982474

Conformer **2A**-d

Center Number	Atomic Number	Atomic Type	Coordinates (Angstroms)		
			X	Y	Z
1	6	0	−3.916605	−1.650912	−0.068704
2	6	0	−4.611745	−0.434799	−0.069578
3	6	0	−3.921019	0.789943	−0.009173
4	6	0	−2.542579	0.731891	0.057893
5	6	0	−1.814215	−0.467669	0.081709
6	6	0	−2.513522	−1.660554	0.007461
7	7	0	−1.629008	1.789857	0.096863
8	6	0	−0.310699	1.386566	0.235278
9	6	0	−0.320192	−0.170085	0.187683
10	6	0	0.233648	−0.785539	1.507095

11	6	0	1.754997	−1.015768	1.65708
12	7	0	2.406393	0.217729	2.102493
13	6	0	3.312658	0.754455	1.392123
14	6	0	3.847646	0.259668	0.085788
15	6	0	2.968618	−0.767072	−0.60071
16	6	0	2.422238	−1.743815	0.457406
17	6	0	1.497488	−2.745842	−0.236815
18	8	0	0.467388	−2.145921	−1.034221
19	6	0	0.427402	−0.716366	−1.08311
20	6	0	1.80821	−0.114332	−1.400575
21	6	0	5.316691	0.374098	−0.123025
22	8	0	4.41846	1.303434	−0.735555
23	8	0	0.631959	2.150067	0.340574
24	6	0	6.137117	−0.508492	−1.030838
25	8	0	−2.037486	3.09316	0.302384
26	6	0	−1.75704	3.904775	−0.853273
27	8	0	−5.971009	−0.322981	−0.130267
28	6	0	−6.743277	−1.511607	−0.182814
29	1	0	−4.448436	−2.592908	−0.125065
30	1	0	−4.464663	1.726985	−0.007548
31	1	0	−1.982141	−2.606906	−0.004256
32	1	0	−0.251777	−1.760267	1.615873
33	1	0	−0.096647	−0.180965	2.358164
34	1	0	1.833574	−1.693127	2.519803
35	1	0	3.761809	1.685186	1.752435
36	1	0	3.573969	−1.35495	−1.29789
37	1	0	3.269499	−2.316701	0.859385
38	1	0	2.097144	−3.382922	−0.903177
39	1	0	1.016728	−3.405619	0.499241
40	1	0	−0.229295	−0.504822	−1.932648
41	1	0	1.988047	−0.270734	−2.469173
42	1	0	1.786718	0.964401	−1.236695
43	1	0	5.884678	0.806866	0.706018
44	1	0	6.968062	0.068831	−1.452509
45	1	0	6.56331	−1.349291	−0.470505
46	1	0	5.547296	−0.899511	−1.863411
47	1	0	−2.160056	4.888121	−0.601725
48	1	0	−0.678889	3.970398	−1.019368
49	1	0	−2.262724	3.507234	−1.740608
50	1	0	−7.785605	−1.189064	−0.216054
51	1	0	−6.517366	−2.101267	−1.081113
52	1	0	−6.584351	−2.135418	0.706599

Table S15. Standard orientations of configurations **2B** for calculations.

Conformer **2B** -a

Center Number	Atomic Number	Atomic Type	Coordinates (Angstroms)		
			X	Y	Z
1	6	0	−3.830023	−1.89885	−0.131151
2	6	0	−4.522988	−0.679495	−0.200299
3	6	0	−3.832139	0.543164	−0.166717
4	6	0	−2.449173	0.471274	−0.061853

5	6	0	−1.731537	−0.723915	0.007287
6	6	0	−2.437962	−1.922307	−0.026277
7	7	0	−1.532968	1.530999	−0.008576
8	6	0	−0.211641	1.116664	−0.049197
9	6	0	−0.236943	−0.431467	0.112497
10	6	0	0.251035	−0.872958	1.524796
11	6	0	1.76702	−0.987215	1.792585
12	7	0	2.310907	0.319319	2.170706
13	6	0	3.159233	0.89434	1.419952
14	6	0	3.711827	0.392308	0.126398
15	6	0	3.060125	−0.859981	−0.439458
16	6	0	2.568861	−1.760948	0.713196
17	6	0	1.79082	−2.939479	0.124262
18	8	0	0.663639	−2.560699	−0.681754
19	6	0	0.553197	−1.175657	−1.023641
20	6	0	1.9084	−0.590221	−1.448413
21	6	0	4.474564	1.346778	−0.706935
22	8	0	5.172518	0.420487	0.132807
23	8	0	0.73748	1.873608	−0.161725
24	6	0	4.671753	1.213228	−2.197836
25	8	0	−1.911189	2.825251	−0.316499
26	6	0	−1.723542	3.690277	0.818656
27	8	0	−5.879971	−0.780268	−0.303982
28	6	0	−6.641015	0.413843	−0.386659
29	1	0	−4.405406	−2.817836	−0.167783
30	1	0	−4.328275	1.502303	−0.234265
31	1	0	−1.908363	−2.869409	0.006436
32	1	0	−0.18732	−1.860414	1.696114
33	1	0	−0.176513	−0.207141	2.282113
34	1	0	1.829589	−1.583691	2.714025
35	1	0	3.555922	1.857855	1.754512
36	1	0	3.851585	−1.410515	−0.958438
37	1	0	3.455347	−2.174457	1.212422
38	1	0	2.470145	−3.541296	−0.498064
39	1	0	1.41033	−3.593214	0.920082
40	1	0	−0.096293	−1.169469	−1.904545
41	1	0	1.797057	0.477141	−1.643635
42	1	0	2.156532	−1.069917	−2.401907
43	1	0	4.516051	2.377502	−0.342887
44	1	0	5.664729	1.587245	−2.473101
45	1	0	3.926003	1.808136	−2.737106
46	1	0	4.598972	0.174349	−2.528666
47	1	0	−2.312359	3.34313	1.675156
48	1	0	−0.664119	3.750881	1.080297
49	1	0	−2.085279	4.665042	0.484102
50	1	0	−7.683599	0.099356	−0.461083
51	1	0	−6.513706	1.036949	0.508627
52	1	0	−6.375654	1.001892	−1.27524

Conformer **2B** -b

Center Number	Atomic Number	Atomic Type	Coordinates (Angstroms)		
			X	Y	Z

1	6	0	−3.972462	−1.479198	−0.104195
2	6	0	−4.553278	−0.208039	−0.20107
3	6	0	−3.75551	0.952116	−0.19268
4	6	0	−2.390423	0.774946	−0.082259
5	6	0	−1.775755	−0.482069	0.016494
6	6	0	−2.577801	−1.610612	0.005842
7	7	0	−1.383231	1.747991	−0.053695
8	6	0	−0.102956	1.219803	−0.074779
9	6	0	−0.26181	−0.316744	0.118826
10	6	0	0.186707	−0.767724	1.541108
11	6	0	1.688267	−0.999105	1.815119
12	7	0	2.337793	0.265339	2.166919
13	6	0	3.22902	0.753161	1.404312
14	6	0	3.735459	0.182424	0.120455
15	6	0	2.985782	−1.026346	−0.418405
16	6	0	2.422791	−1.858976	0.753117
17	6	0	1.550585	−2.982491	0.188942
18	8	0	0.457511	−2.53152	−0.626794
19	6	0	0.461841	−1.149823	−0.999912
20	6	0	1.860378	−0.689408	−1.437146
21	6	0	4.566712	1.058653	−0.732913
22	8	0	5.193964	0.096821	0.122483
23	8	0	0.909839	1.887647	−0.194231
24	6	0	4.747293	0.884503	−2.221499
25	8	0	−1.643034	3.065531	−0.379855
26	6	0	−1.413863	3.922347	0.753899
27	8	0	−5.894233	0.02066	−0.312813
28	6	0	−6.767616	−1.09675	−0.348089
29	1	0	−4.58647	−2.371473	−0.11779
30	1	0	−4.210359	1.930952	−0.285535
31	1	0	−2.130327	−2.598345	0.060045
32	1	0	−0.329776	−1.712944	1.731013
33	1	0	−0.186451	−0.054349	2.283775
34	1	0	1.70125	−1.579597	2.748819
35	1	0	3.704538	1.687252	1.718833
36	1	0	3.731389	−1.649621	−0.923014
37	1	0	3.272936	−2.333089	1.26171
38	1	0	2.178165	−3.651388	−0.419072
39	1	0	1.118642	−3.584998	0.998859
40	1	0	−0.186129	−1.109289	−1.881005
41	1	0	1.837018	0.37777	−1.661049
42	1	0	2.068379	−1.213115	−2.377064
43	1	0	4.688786	2.089622	−0.388222
44	1	0	5.762508	1.183823	−2.506957
45	1	0	4.042156	1.520063	−2.768994
46	1	0	4.601851	−0.152626	−2.533616
47	1	0	−2.051057	3.632426	1.596828
48	1	0	−0.359766	3.899018	1.042563
49	1	0	−1.68646	4.920159	0.403441
50	1	0	−7.774109	−0.685767	−0.445815
51	1	0	−6.556034	−1.747241	−1.206871

52	1	0	−6.705172	−1.687668	0.57524
Conformer **2B** -c					
Center Number	Atomic Number	Atomic Type	Coordinates (Angstroms)		
			X	Y	Z
1	6	0	−3.832682	−1.871366	−0.300855
2	6	0	−4.53766	−0.666251	−0.150775
3	6	0	−3.85607	0.5439	0.059624
4	6	0	−2.470089	0.472949	0.115735
5	6	0	−1.741176	−0.711053	−0.008767
6	6	0	−2.438582	−1.894916	−0.228165
7	7	0	−1.560575	1.524658	0.282249
8	6	0	−0.238966	1.119472	0.33147
9	6	0	−0.248736	−0.424476	0.137515
10	6	0	0.278604	−1.168556	1.400099
11	6	0	1.799725	−1.362813	1.586977
12	7	0	2.385432	−0.191088	2.240165
13	6	0	3.243963	0.517226	1.628312
14	6	0	3.763925	0.310003	0.242762
15	6	0	3.047227	−0.746021	−0.583082
16	6	0	2.556694	−1.882145	0.336006
17	6	0	1.73823	−2.873857	−0.492485
18	8	0	0.621853	−2.290558	−1.183111
19	6	0	0.520645	−0.865195	−1.16018
20	6	0	1.874562	−0.19333	−1.441141
21	6	0	4.555692	1.402725	−0.362818
22	8	0	5.22341	0.278601	0.22121
23	8	0	0.706878	1.877137	0.470966
24	6	0	4.728046	1.614893	−1.847716
25	8	0	−1.971781	2.805406	0.592414
26	6	0	−1.717327	3.704832	−0.503099
27	8	0	−5.896303	−0.76694	−0.227726
28	6	0	−6.670158	0.411903	−0.074747
29	1	0	−4.400884	−2.779709	−0.470996
30	1	0	−4.362712	1.491925	0.183109
31	1	0	−1.902053	−2.829856	−0.355424
32	1	0	−0.173273	−2.164359	1.369789
33	1	0	−0.110053	−0.680262	2.300035
34	1	0	1.871303	−2.156152	2.344836
35	1	0	3.673744	1.366172	2.168644
36	1	0	3.796046	−1.17873	−1.254725
37	1	0	3.442451	−2.419292	0.700859
38	1	0	2.394	−3.339954	−1.242812
39	1	0	1.342587	−3.678287	0.141785
40	1	0	−0.140832	−0.632495	−2.000921
41	1	0	1.775019	0.886431	−1.317502
42	1	0	2.093748	−0.378851	−2.498555
43	1	0	4.647469	2.316734	0.231263
44	1	0	5.729851	2.011401	−2.049851
45	1	0	3.997087	2.342614	−2.218417
46	1	0	4.614609	0.684675	−2.409849
47	1	0	−0.642821	3.789158	−0.683159

48	1	0	−2.236847	3.37234	−1.409122
49	1	0	−2.120217	4.663893	−0.170545
50	1	0	−7.71211	0.099292	−0.16428
51	1	0	−6.513236	0.87376	0.908986
52	1	0	−6.444536	1.147851	−0.858093

Conformer **2B** -d

Center Number	Atomic Number	Atomic Type	Coordinates (Angstroms)		
			X	Y	Z
1	6	0	−3.972952	−1.462379	−0.235023
2	6	0	−4.56929	−0.203895	−0.082579
3	6	0	−3.783007	0.947935	0.110095
4	6	0	−2.413375	0.774068	0.146623
5	6	0	−1.783377	−0.47283	0.017742
6	6	0	−2.574776	−1.591423	−0.181505
7	7	0	−1.415426	1.743445	0.295248
8	6	0	−0.131216	1.230696	0.320141
9	6	0	−0.270999	−0.309705	0.146708
10	6	0	0.200941	−1.07857	1.416517
11	6	0	1.702111	−1.391751	1.600141
12	7	0	2.382807	−0.262998	2.237134
13	6	0	3.289805	0.36906	1.611537
14	6	0	3.782053	0.106469	0.22525
15	6	0	2.983209	−0.903558	−0.583102
16	6	0	2.409881	−1.984845	0.354457
17	6	0	1.511913	−2.920524	−0.456879
18	8	0	0.438544	−2.261111	−1.14785
19	6	0	0.451987	−0.831432	−1.147794
20	6	0	1.853255	−0.275338	−1.446137
21	6	0	4.647826	1.129791	−0.400101
22	8	0	5.235081	−0.033374	0.193838
23	8	0	0.876625	1.909005	0.429626
24	6	0	4.821798	1.309049	−1.889187
25	8	0	−1.708699	3.058289	0.592269
26	6	0	−1.432698	3.912638	−0.533702
27	8	0	−5.915379	0.019812	−0.108662
28	6	0	−6.779538	−1.090934	−0.288354
29	1	0	−4.578448	−2.346742	−0.39217
30	1	0	−4.250673	1.917689	0.230613
31	1	0	−2.11849	−2.568218	−0.309042
32	1	0	−0.329313	−2.035284	1.398884
33	1	0	−0.143869	−0.550579	2.311916
34	1	0	1.71322	−2.179037	2.367573
35	1	0	3.788414	1.187858	2.139119
36	1	0	3.694574	−1.401936	−1.249858
37	1	0	3.252658	−2.584634	0.723408
38	1	0	2.124709	−3.445233	−1.204962
39	1	0	1.059884	−3.684897	0.189166
40	1	0	−0.194045	−0.560014	−1.988951
41	1	0	1.840091	0.810589	−1.340992
42	1	0	2.050951	−0.496235	−2.50105
43	1	0	4.81179	2.042067	0.180824

44	1	0	5.849575	1.622745	−2.105794
45	1	0	4.146534	2.087333	−2.26279
46	1	0	4.629082	0.383692	−2.437501
47	1	0	−0.363279	3.910408	−0.759351
48	1	0	−2.014548	3.603304	−1.409369
49	1	0	−1.746923	4.906387	−0.207759
50	1	0	−7.793163	−0.686364	−0.269871
51	1	0	−6.604363	−1.587252	−1.251955
52	1	0	−6.666722	−1.824877	0.520385

4.3. Experimental and computed NMR chemical shifts

4.3.1. ^{13}C-NMR chemical shifts

Table 13. C-NMR chemical shifts of compounds **2A** and **2B** were fitted to the experimental values by Ordinary Least Squares (OLS) Linear Regression method in order to remove systematic error that results from the conformational search and random error from experimental conditions (**Error! Reference source not found.** and **S17**).

Table S16. Experimental and computed chemical shifts of **2A**.

^{13}C-NMR:

Position	Experimental	Calculated-2A	Fitted-2A
2	174.47	180.2132	174.9287
3	74.95	78.48834	75.64154
5	60.35	64.75028	62.2327
6	37.05	41.00305	39.05455
7	57.44	61.59328	59.15136
8	124.83	129.7347	125.6598
9	126.99	131.0379	126.9318
10	109.29	110.1695	106.5635
11	162.09	166.383	161.4299
12	95.47	96.21604	92.94442
13	140.65	145.8977	141.4356
14	25.47	26.54152	24.93957
15	25.15	28.18716	26.54577
16	34.22	37.25172	35.39312
17	67.8	69.77088	67.13299
18	13.89	15.05666	13.72993
19	60.10	63.18715	60.70702
20	62.35	64.6732	62.15746
21	167.57	174.3352	169.1915
NOMe	64.17	63.88062	61.38388
ArOMe	56.21	55.65453	53.35491

Table S17. Experimental and computed chemical shifts of **2B**.

^{13}C-NMR:

Position	Experimental	Calculated-2B	Fitted-2B
2	174.47	180.3253023	175.8351
3	74.95	79.20651849	76.22454
5	60.35	64.63984776	61.87514
6	37.05	40.22039725	37.81992
7	57.44	61.58278025	58.86367
8	124.83	129.833446	126.0963
9	126.99	130.5828925	126.8346
10	109.29	110.3398794	106.8935
11	162.09	166.393335	162.1109
12	95.47	96.23624678	93.00026
13	140.65	145.6206905	141.6481
14	25.47	28.42820989	26.20362
15	25.15	33.13623033	30.84142
16	34.22	35.55256475	33.22171
17	67.8	69.10364915	66.27236
18	13.89	16.28785209	14.24435
19	60.10	64.05971138	61.30366
20	62.35	61.92021982	59.19608
21	167.57	172.3178368	167.9471
NOMe	64.17	63.79167934	61.03963
ArOMe	56.21	55.66887491	53.03798

Relatively higher R^2 and lower CMAD and CLAD values were shown in both ^{13}C-NMR Ordinary Least Squares Linear Regression (OLS-LR) for compound **2**, which indicated that this configuration was the correct structure (**Table S18**).

Table S18. Statistics of Ordinary Least Squares Linear Regression (OLS-LR) of experimental and computed ^{13}C-NMR chemical shifts.

Type	Compound	CMAD[a]	CLAD[b]	R^2	*RMSD*	*F*	*p* value
^{13}C NMR	**2A**	1.25	2.86	0.9990	1.6184	18704.19	< 0.01
	**2B**	1.62	5.69	0.9982	2.1841	10255.04	< 0.01

[a] CMAD = corrected mean absolute deviation, computed as $(1/n)\sum_{i}^{n}|\delta_{calc}-\delta_{exp}|$, where δ_{calc} and δ_{exp} refer to the calculated and experimental chemical shifts. [b] CLAD = corrected largest absolute deviation, computed as $\max(|\delta_{calc}-\delta_{exp}|)$.

References

19. O'Boyle, N.M.; Vandermeersch, T.; Flynn, C.J.; Maguire, A.R.; Hutchison, G.R. Confab-Systematic generation of diverse low-energy conformers. *J. cheminformatics*, **2011**, *3*, 1–9.
20. Frisch, M. J.; Trucks, G. W.; Schlegel, H. B.; Scuseria, G. E.; Robb, M. A.; Cheeseman, J. R.; Scalmani, G.; Barone, V.; Mennucci, B.; Petersson, G. A.; Nakatsuji, H.; Caricato, M.; Li, X.; et al. Gaussian 09 Revision D.01. Gaussian Inc.: Wallingford, CT USA, 2009.
21. Lodewyk, M.W.; Siebert, M.R.; Tantillo, D.J. Computational prediction of 1H and 13C chemical shifts: a useful tool for natural product, mechanistic, and synthetic organic chemistry. *Chem. Rev.* , **2012**, *112*, 1839–1862.
